# Liver Metastases from Colorectal Cancer: From Pathogenesis to Current Treatment

**DOI:** 10.1002/mco2.71044

**Published:** 2026-10-04

**Authors:** Jialiang Fan, Huiru Zhang, Deyang Kong, Yu Sun, Shuaibing Lu, Wenjing Yang, Renshen Xiang, Qi Zhang, Wei Pei, Haizeng Zhang, Lin Feng

**Affiliations:** ^1^ Department of Etiology and Carcinogenesis National Cancer Center/National Clinical Research Center for Cancer/Cancer Hospital Chinese Academy of Medical Sciences and Peking Union Medical College Beijing China; ^2^ State Key Laboratory of Molecular Oncology National Cancer Center/National Clinical Research Center for Cancer/Cancer Hospital Chinese Academy of Medical Sciences and Peking Union Medical College Beijing China; ^3^ Department of Colorectal Surgery National Cancer Center/National Clinical Research Center for Cancer/Cancer Hospital Chinese Academy of Medical Sciences and Peking Union Medical College Beijing China

**Keywords:** biomarkers, colorectal cancer, liver metastasis, pathogenesis, tumor microenvironment

## Abstract

Colorectal cancer (CRC) remains one of the most frequently diagnosed malignancies worldwide, and colorectal cancer liver metastasis (CRLM) represents a major cause of CRC‐related mortality. The liver provides a highly specialized environment that can be remodeled into a metastatic niche, where anatomical structure, metabolic properties, and immunological landscape collectively influence tumor cell survival, colonization, and outgrowth. Increasing evidence indicates that CRLM development is driven by dynamic interactions between tumor intrinsic programs and the hepatic tumor microenvironment(TME), involving epithelial plasticity, intercellular communication, stromal remodeling, and immune regulation. However, an integrated understanding that links metastatic initiation, hepatic niche formation, immune escape, and therapeutic intervention remain lacking. This review examines recent advances in the multistage progression of CRLM and discusses how immune tolerance, lymphocyte dysfunction, and suppressive cellular networks contribute to the establishment and maintenance of the CRLM. Finally, we review current clinical management strategies and emerging approaches, immunotherapy, and biomarker‐guided precision treatment. By integrating mechanistic insights with clinical perspectives, this review aims to provide a conceptual framework for understanding CRLM progression and to identify potential directions for improving individualized therapeutic strategies.

## Graphical abstract

The liver is not merely a passive recipient of disseminated tumor cells, but a unique metastatic organ shaped by anatomical, metabolic, and immunological characteristics that support tumor colonization. Increasing evidence indicates that colorectal cancer liver metastasis development is driven by dynamic interactions between tumor intrinsic programs and the hepatic tumor microenvironment.

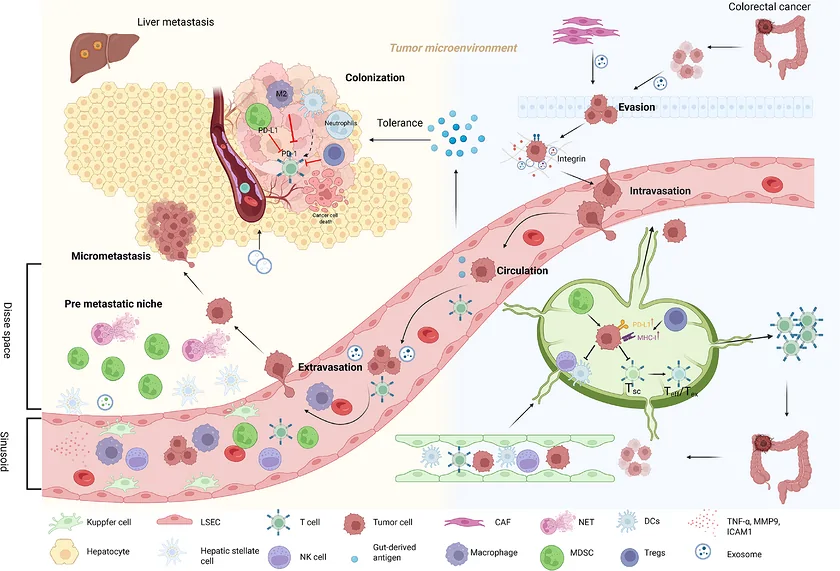

## 1 Introduction

Colorectal cancer (CRC) remains a major global health challenge, ranking as the third most common malignant tumor worldwide [1]. The liver is the most frequent site of metastasis in patients with CRC, and the development of colorectal cancer liver metastasis (CRLM) significantly influences therapeutic decision‐making and clinical outcomes [2]. Approximately 20–30% of individuals with CRC eventually develop CRLM during the course of disease [3]. Although advances in early detection, surgery and systemic therapy have improved outcomes for patients with early‐stage disease, individuals with CRLM still face high rates of recurrence and mortality [4]. Therefore, understanding why CRC preferentially colonizes the liver and how metastatic lesions are established and persist within this organ is essential for developing more effective therapeutic strategies.

Accumulating evidence indicates that CRLM is not simply a consequence of passive tumor cell dissemination but rather a complex evolutionary process involving continuous interactions between tumor cells and the hepatic microenvironment. The tumor microenvironment (TME), which is composed of immune cells, stromal cells, the extracellular matrix (ECM), and vascular components, actively influences multiple stages of metastasis, from tumor cell invasion and survival in circulation, hepatic colonization and therapeutic response. Concurrently, intrinsic tumor programs such as epithelial–mesenchymal transition (EMT), metabolic adaptation, and stemness acquisition cooperate with host‐derived signals to reshape the metastatic niche. In particular, the liver itself provides a distinct metastatic context characterized by portal venous circulation, specialized immune regulation, and complex stromal organization, which together shape a microenvironment that can support the survival and expansion of CRC‐derived metastatic cells [5, 6, 7].

Given the pivotal contribution of the liver microenvironment to CRC metastasis, understanding the molecular and cellular mechanisms governing its formation and remodeling is essential for developing more effective antimetastatic strategies. In this review, we provide a comprehensive overview of the biological continuum from CRC development to liver metastatic progression. We first discuss the determinants of liver tropism, including metastatic evolution, circulating tumor cells (CTCs) survival, EMT plasticity, hepatic premetastatic niche (PMN) formation, and the gut–liver axis. We then focus on the dynamic remodeling of the immune microenvironment in CRLM, highlighting the functional roles of lymphocyte populations, immune tolerance mechanisms, and cellular interactions that facilitate metastatic persistence. Finally, we summarize current clinical management strategies and emerging TME‐targeted, immunotherapy, and precision biomarker‐guided treatment approaches. By integrating mechanistic insights with clinical perspectives, this review aims to establish an integrated framework for understanding CRLM pathogenesis and to provide directions for the future development of personalized therapies.

## 2 From Primary Colorectal Tumors to Liver Metastases: Tumors and Host Determinants

The development of CRLM represents a dynamic evolutionary process rather than a simple consequence of CRC cells transport through the portal circulation. Successful liver colonization requires coordinated changes in CRC cells and the host microenvironment, including metastatic dissemination, survival during circulation, adaptation to hepatic immune tolerance, and interactions with stromal components and gut‐derived signals.

### 2.1 Anatomical and Hemodynamic Basis for Liver Tropism

The liver has a distinctive dual blood supply, receiving blood from both the hepatic artery and the portal vein. Because the portal vein receives venous drainage from the gastrointestinal tract and accounts for the majority of hepatic blood inflow [8], the liver is the first organ encountered by CRC cells disseminating from the intestinal primary tumor [9, 10]. In addition, the low‐pressure sinusoidal microcirculation, fenestrated endothelial structure, and narrow sinusoidal architecture favor the mechanical trapping, adhesion, and retention of circulating CRC cells [11, 12]. These features provide a hemodynamic basis for hepatic seeding and early extravasation. However, vascular delivery alone is insufficient for metastasis formation. Following hepatic arrival, disseminated CRC cells must overcome multiple barriers, including intravascular stress, immune surveillance, and environmental adaptation, before establishing clinically detectable metastatic lesions.

### 2.2 CTC Survival, EMT Plasticity, and Vascular Arrest

After intravasation, disseminated CRC cells are exposed to a series of selective pressures within the circulation, where most of the CTCs are eliminated due to hemodynamic shear stress, anoikis caused by loss of ECM attachment, oxidative stress, and immune‐mediated elimination [13, 14]. However, a small fraction of CTCs engage in acquire adaptive traits that allow them to persist in circulation and retain metastatic potential. Among the mechanisms supporting CTCs survival, interactions between platelets and tumor cells have been extensively investigated. After vascular entry, platelets rapidly coat CTCs, forming a physical shield that protects CRC cells from shear forces and natural killer cells (NK cells)‐mediated cytotoxicity while simultaneously providing adhesion molecules and growth signals that facilitate vascular arrest and extravasation [15]. In CRC, platelet‐derived factors, including transforming growth factor‐β (TGF‐β), have also been shown to promote EMT‐associated phenotypes and enhance metastatic capacity [16].

Furthermore, multicellular CTC clusters exhibit substantially greater metastatic potential than individual CRC cells. Interactions among clustered tumor cells preserve epithelial characteristics, maintain intercellular survival signals, and increase resistance to anoikis and immune‐mediated clearance [17]. Recent single‐cell and genomic analyses have demonstrated that CTC clusters possess distinct transcriptional programs associated with stemness, stress adaptation, and metastatic initiation, suggesting that collective dissemination is not merely an intermediate stage of tumor spread but may represent an important route for CRLM development [18].

EMT plasticity further contributes to the metastatic competence of CRC cells. Rather than undergoing complete epithelial‐to‐mesenchymal conversion, many metastatic CRC cells retain hybrid epithelial–mesenchymal phenotypes characterized by epithelial adhesion and mesenchymal migratory capacity [19]. Such a partial EMT state may provide a functional advantage by supporting collective migration, maintenance of cell–cell junctions, and promoting CTC cluster formation, while avoiding the excessive loss of epithelial properties associated with complete EMT [20]. EMT plasticity not only is involved in dissemination from the primary tumor but also contributes to metastatic colonization and therapeutic resistance after tumor cells arrive at distant organs.

### 2.3 Physiological Hepatic Tolerance as an Immunological Context for Metastatic Colonization

Upon reaching the liver, disseminated CRC cells encounter a highly specialized hepatic microenvironment. As a central metabolic organ, the liver is continuously exposed to dietary antigens, microbial products, and other gut‐derived molecules. To maintain homeostasis under this constant antigenic challenge, the liver possesses a physiologically tolerogenic immune environment that favors controlled immune activation rather than sustained inflammatory responses under physiological conditions [21, 22]. This baseline tolerance is mediated by hepatocytes and liver‐resident antigen‐presenting or immunoregulatory cells, including liver sinusoidal endothelial cells (LSECs), Kupffer cells (KCs), and hepatic stellate cells (HSCs). In the setting of CRLM, however, this physiological tolerance may provide an initial advantage for disseminated CRC cells during hepatic colonization.

These cells within the liver can present antigens, but this activity is often coupled with limited costimulation, inhibitory checkpoint signaling, and immunoregulatory cytokine production [23]. Molecules such as programmed death‐ligand 1 (PD‐L1) and LSCS lectin, together with IL‐10 and TGF‐β, contribute to T‐cell hyporesponsiveness, decrease Th1‐biased responses, promote the differentiation of FoxP3^+^ regulatory T cells (Tregs), and limit excessive T‐cell accumulation in the liver [24, 25, 26]. Thus, hepatic tolerance is not mediated by a single cell type but by a coordinated program of restrained antigen presentation, inhibitory signaling, and regulatory immune responses [27, 28, 29, 30].

While this tolerogenic program is essential for preventing unnecessary inflammation in healthy liver tissue, it may also create a favorable immunological background for metastatic seeding. CRC cells arriving in the liver encounter an organ in which immune activation is tightly controlled, and stromal remodeling can be rapidly induced. During metastatic progression, cells within the liver may be reprogrammed to support suppressive myeloid recruitment, Treg‐rich states, and tumor growth; these established suppressive circuits are discussed in Section 3.

### 2.4 Formation of the Hepatic PMN in CRLM

Before clinically detectable CRLM develops, primary CRC tumors can systemically reshape the hepatic microenvironment, establishing a permissive PMN that facilitates subsequent metastatic seeding. Tumor‐derived extracellular vesicles (EVs), together with cytokines, inflammatory mediators, and metabolic signals circulate to distant organs and initiate a series of adaptive changes within the liver [31]. These alterations affect multiple components of the hepatic ecosystem, including immune suppression, ECM remodeling, vascular dysfunction, and metabolic reprogramming, ultimately converting the liver into an environment that supports metastasis.

Among the diverse tumor‐derived factors, EVs, particularly exosomes, are important mediators of liver preconditioning because they transport functional cargo, including microRNAs, long noncoding RNAs, proteins, and metabolites, to the liver. Several CRC‐derived exosomal miRNAs have been linked to impaired NK cell cytotoxicity and the acquisition of an M2‐like macrophage phenotype, thereby establishing immune conditions favorable for metastatic growth. Mechanistically, exosomal miR‐106a‐5p activates the JAK2/STAT3 pathway via SOCS6 inhibition, whereas miR‐25‐3p, miR‐130b‐3p, miR‐425‐5p, and miR‐934 activate PI3K/AKT signaling by downregulating PTEN expression, collectively facilitating an immunosuppressive macrophage phenotype that supports CRLM progression [32, 33, 34]. In addition to miRNAs, exosome‐associated lncRNAs can remodel immune metabolism, and the lncRNA PWAR6 derived from cancer‐associated fibroblasts (CAFs) reduces glutamine availability to NK cells by upregulating SLC38A2 expression through Keap1–NRF2 signaling, thereby promoting immune evasion [35].

CRC‐derived exosomes also reprogram stromal and metabolic circuits in the liver. Exosomal miR‐1246 activates HSCs by targeting INSIG1, leading to SREBP2‐mediated cholesterol accumulation and activation of the TLR4/NF‐κB/TGF‐β pathway [36]. Similarly, the exosomal lncRNA MIR181A1HG promotes HSCs activation via the TGFβRII/Smad2/3 pathway [36]. Activated HSCs subsequently promote ECM remodeling and foster an immunosuppressive milieu through the recruitment of myeloid‐derived suppressor cells (MDSCs), contributing to CRLM via the TNFSF13/TNFRSF13B axis [36, 37]. KCs may further amplify prometastatic remodeling by selectively internalizing circulating exosomal cargo: uptake of miR‐135a‐5p from circulating CRC cells activates the LATS2–YAP–MMP7 axis, which increases metastatic CRC cell adhesion and suppresses CD4^+^ T‐cell responses [38]. Together, these studies support a model in which exosomal signals initiate coordinated immune and stromal conditioning of the liver before metastatic colonization.

Vascular remodeling represents another component of PMN formation. Increased angiogenesis and vascular permeability can facilitate tumor cell arrest, extravasation, and survival within the liver, and exosomes provide one mean by which CRC cells communicate with the vascular endothelium [39]. Circulating CRC cells, for example, release exosomal miR‐1825, which promotes angiogenesis through activation of TGF‐β/Smad2/3 signaling [40], whereas miR‐25‐3p disrupts endothelial integrity by downregulating the expression of tight junction proteins by targeting KLF2/4 [41]. In addition to noncoding RNAs, exosomes contain proteins and other bioactive molecules that participate in stromal activation, immune suppression, angiogenesis, and metabolic remodeling. Representative exosomal cargoes implicated in CRLM are summarized in Table 1.

**TABLE 1:** Non‐RNA extracellular vesicles components involved in the formation of the premetastatic niche in colorectal cancer liver metastasis.

Exosome component	Source	Major effect in CRLM‐related PMN formation	References
ADAM17	Tumor	Promotes CRC‐cell migration	[42]
AMIGO2	Tumor	Activates HSCs and induces IL‐8 secretion	[43]
ANXA1	Tumor	Promotes angiogenesis and M2‐like macrophage polarization	[44]
ANGPTL1	Tumor	Limits vascular leakiness and suppresses metastasis	[45]
GRP78	Tumor	Inhibits DC activation, promotes M2‐like macrophage polarization, and enhances TGF‐β production	[46]
HSP90B1	Tumor	Regulates M2 macrophage polarization and reduces CD8^+^ T‐cell viability	[47]
HSPC111	Tumor	Reprograms lipid metabolism in CAFs	[48]
ITGBL1	Tumor	Activates fibroblasts through NF‐κB signaling	[49]
tRF‐GluCTC‐0005	Tumor	Activates HSCs and mediates the infiltration of MDSCs	[50]
TGFβ1	Tumor	Promotes hepatocyte EMT	[51]
TGFβ1	Tumor	Induces NK‐cell dysfunction	[52]

Abbreviations: HSCs: hepatic stellate cells; DCs: dendritic cells; CAFs: cancer‐associated fibroblasts; MDSCs: myeloid‐derived suppressor cells; EMT: epithelial–mesenchymal transition; NK: natural killer cells.

Increasing evidence suggest that in addition to tumor‐derived exosomal signals, the gut–liver axis represents an additional route through which the hepatic PMN is established. The liver is continuously exposed to intestinally derived factors through the portal circulation, making it particularly sensitive to changes in gut barrier integrity and microbial composition. Experimental evidence indicates that impairment of the gut vascular barrier can permit bacterial translocation to the liver and create conditions that favor subsequent CRLM. In CRC, tumor‐associated alterations in intestinal permeability may result in the liver being exposed to microbial products, including lipopolysaccharides, bacterial DNA, and other pathogen‐associated molecular patterns. These signals can activate hepatic myeloid cells through pattern recognition receptors, particularly Toll‐like receptor, leading to inflammatory cytokine production, ECM remodeling, and the recruitment of immunosuppressive myeloid cells that support metastatic colonization [10, 53].

In addition to direct microbial translocation, metabolites produced or modified by the gut microbiota can influence the susceptibility of the liver to metastatic colonization. Bile acids (BAs), which are extensively modified by intestinal bacteria, function as signaling molecules that regulate immune cell differentiation, epithelial integrity, and inflammatory responses through pathways involving FXR and TGR5 [54]. Disruption of BA metabolism in CRC can reshape the hepatic immune landscape and promote the recruitment of metastasis‐associated neutrophils. Microbiota‐driven alterations in secondary BA production increase CXCL5/CXCR2‐dependent neutrophil infiltration, resulting in the establishment of an inflammatory hepatic niche that facilitates CRLM [55].

Collectively, CRC‐derived exosomes, soluble mediators, and gut–liver axis signals cooperate to induce immune suppression, stromal activation, metabolic reprogramming, and vascular remodeling, thereby generating a hepatic PMN that supports CRC cell seeding and outgrowth. These processes are summarized in Figure 1.

**FIGURE 1:**
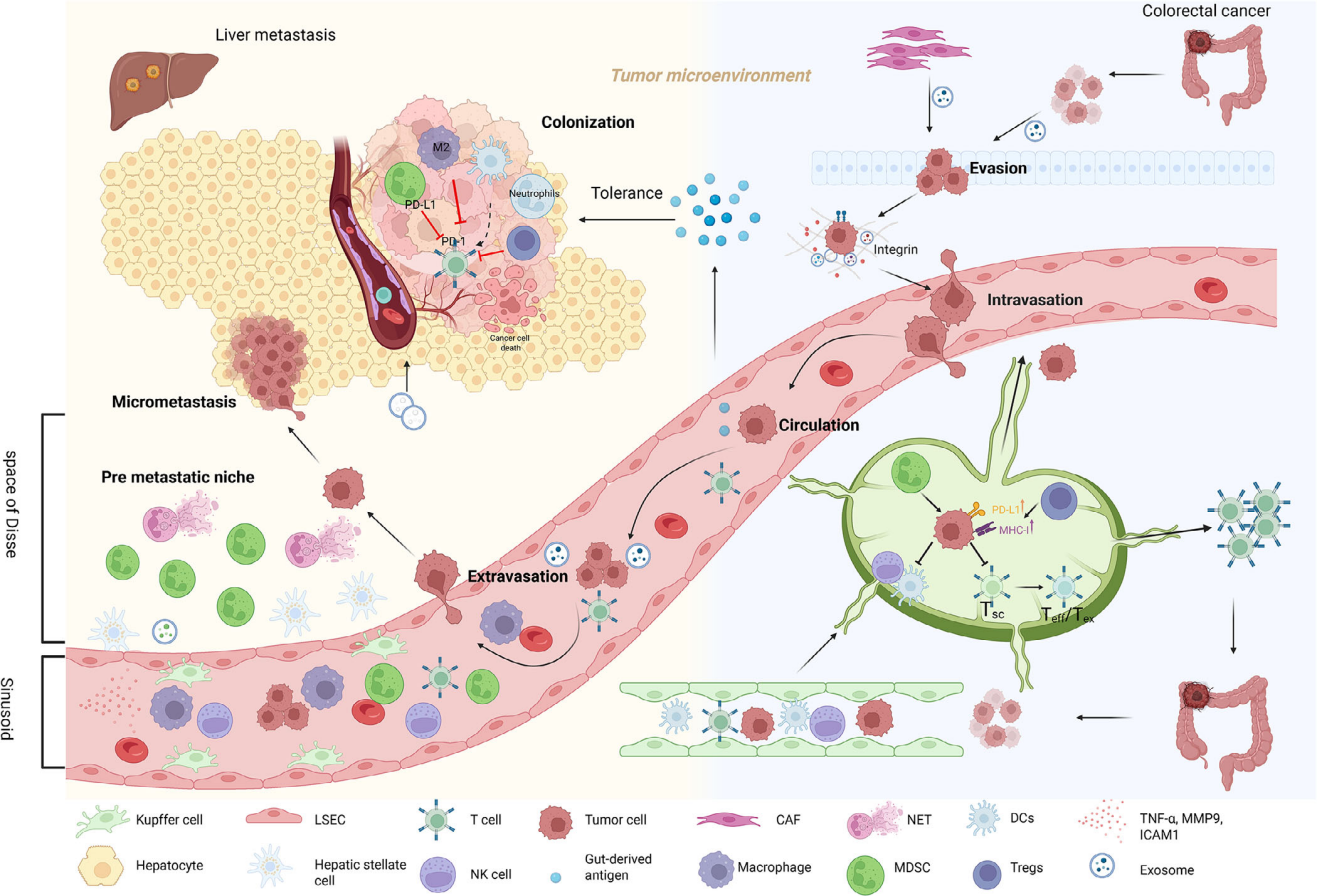
The liver: a preferred target of colorectal cancer metastasis. The unique anatomy and immunosuppressive microenvironment of the liver promote the colonization and survival of disseminated tumor cells from primary CRC. CRC cells spread through the portal vein or tumor‐draining lymph nodes and establish micro‐metastases by interacting with liver‐resident cells such as hepatocytes, Kupffer cells, and LSECs. These cells contribute to a tolerogenic environment that promotes immune evasion via mechanisms such as PD‐L1/PD‐1 signaling and macrophage polarization toward the M2 phenotype. In the premetastatic niche, CRC‐derived exosomes play pivotal roles by reprogramming liver‐resident cells, impairing immune surveillance, and enhancing angiogenesis. These processes collectively promote metastatic outgrowth by creating a favorable “seed and soil” microenvironment in the liver, facilitating invasion, extravasation, and the establishment of a metastatic niche. Immune modulation, stromal remodeling, and metabolic reprogramming play critical roles in CRLM progression, as demonstrated by tumor–exosome interactions with immune and stromal components. NET: neutrophil extracellular trap; MDSCs: myeloid‐derived suppressor cells; NK: natural killer cells; Tregs: regulatory T cells; CAF: cancer‐associated fibroblast; DCs: dendritic cells; LSECs: liver sinusoidal endothelial cells.

### 2.5 Primary Tumor Features Associated with Liver‐tropic Metastasis

CRLM is not only determined by anatomical dissemination through the portal circulation or the permissive hepatic microenvironment. It also reflects evolutionary processes within the primary CRC that determine which cell populations are capable of completing the metastatic cascade. Primary tumors consist of multiple genetically and epigenetically distinct subpopulations that evolve under selective pressures, including hypoxia, nutrient deprivation, immune surveillance, therapeutic stress, and spatial constraints. Within this heterogeneous ecosystem, only a fraction of CRC cells acquire the coordinated traits required for metastatic success, such as invasive capacity, epithelial plasticity, resistance to anoikis, stem‐like properties, and adaptability to the hepatic microenvironment [56]. From this perspective, CRLM represents the outcome of an evolutionary filtering process in which metastatic competent clones are selected from a diverse primary tumor population.

Genomic heterogeneity provides one substrate for this selection. Multiregion sequencing studies have demonstrated that metastatic progression involves complex clonal evolution patterns. Early truncal alterations, including APC, TP53, and KRAS mutations, are frequently maintained across primary CRC and metastatic lesions, indicating their fundamental role in tumorigenesis and their metastatic potential. In contrast, additional branch‐specific alterations emerge during tumor evolution and may provide selective advantages related to dissemination, survival during circulation, immune escape, and the colonization of distant organs [57].

Phylogenetic comparisons across primary and metastatic lesions suggest that CRLM can arise through different dissemination routes. Liver lesions may be seeded directly from the primary tumor by monoclonal or oligoclonal lineages. However, secondary reseeding events, including dissemination through lymph node metastases and reseeding between liver metastases, have also been reported [58]. These findings suggest that CRLM evolution is highly dynamic and influenced by both the intrinsic diversity of tumor subclones and the selective pressures encountered during metastatic dissemination.

At the pathway level, CRLM‐associated evolutionary programs involve coordinated alterations in several signaling networks. Dysregulation of WNT/β‐catenin signaling remains a central feature of CRC progression, while additional alterations affecting the PI3K/AKT, TGF‐β, and receptor tyrosine kinase pathways contribute to survival, invasion, and adaptation within the hepatic environment [59].

## 3 Immune Ecosystem Reprogramming in Established CRLM

Effective antitumor immunity in CRLM requires coordination among tumor antigen recognition, immune cell activation, cytotoxic execution, and local immune organization. The immune dysfunction in CRLM reflects concurrent impairment of tumor recognition, cytotoxic effector function, and the spatial organization required to maintain effective local antitumor immunity.

### 3.1 Failure of Tumor Recognition and Antigen Dependent Immune Activation

One of the earliest barriers to effective immune responses in CRLM is impaired tumor recognition. Cytotoxic CD8^+^ T cells recognize tumor‐derived peptides presented by major histocompatibility complex class I (MHC‐I) molecules, thereby triggering antigen specific cytotoxic responses. In clinical studies, increased infiltration of activated CD8^+^ T cells is associated with improved prognosis in CRC, whereas impaired antigen presentation contributes to immune escape and CRLM [60, 61]. However, liver metastatic lesions frequently display reduced antigen‐presenting capacity, limiting effective recognition by cytotoxic lymphocytes [62]. In CRLM, the prognostic value of immune infiltration is strongly influenced by tumor cell MHC‐I expression, suggesting that T‐cell abundance alone is insufficient to predict effective antitumor immunity unless antigen presentation remains intact.

Tumor‐intrinsic mechanisms can actively establish this immune‐invisible state. CEMIP overexpression in CRC promotes MHC‐I internalization and lysosomal degradation, thereby reducing surface MHC‐I expression and restricting CD8^+^ T‐cell antitumor activity [63]. These findings indicate that antigen presentation deficiency in CRC is not merely a consequence of tumor progression but also an actively regulated immune evasion program. Furthermore, developmental plasticity programs can generate immune‐resistant tumor states. SOX17 was recently identified as a transcriptional regulator of immune evasion in early CRC. Early CRC cells upregulate the expression of the transcription factor SOX17, which downregulates the expression of interferon gamma (IFN‐γ) receptors. This impairs CD8^+^ T‐cell infiltration and limits the tumor's ability to upregulate MHC‐I expression while also inducing a fetal‐like program with loss of LGR5 expression. Fetal and embryonic cells are inherently less immunogenic and exhibit immune privilege, allowing CRC cells to exploit this program to escape immune surveillance. This transition from immunogenic LGR5^+^ cells to immune‐silent LGR5^−^ cells enhances immune evasion [64].

Recognition failure is reinforced during hepatic colonization. Metastatic CRC cells with stemness signatures exhibit highly activated oncogenic pathways, alternative telomere lengthening phenotypes, and reduced MHC‐I expression [65, 66]. Notably, comparisons of primary CRC lesions and metastatic liver lesions have consistently revealed significantly lower MHC‐I expression in patients with CRLM [66]. In particular, CRLM cells mimic several liver cells throughout liver development. These cells exhibit moderate epigenetic activity of MHC and IFN‐responsive genes, further weakening CD8^+^ T‐cell recognition of tumor cells [67]. These findings suggest that CRLM cells undergo not only genetic selection but also phenotypic adaptation to minimize immune detection within the liver.

Immune escape in CRLM is not restricted to impaired CD8^+^ T cell recognition. NK cells normally eliminate MHC‐I‐low tumor cells through missing‐self recognition; however, metastatic CRC cells can simultaneously activate inhibitory NK cell pathways. Liver‐derived fibrinogen‐like protein 1 (FGL1), for example, suppresses both CD8^+^ T cells and NK cells through LAG‐3 signaling and promotes metastatic progression [68]. In addition, engagement of the HLA‐E/CD94–NKG2A inhibitory axis enables tumor cells to suppress NK cell and cytotoxic T‐cell activity despite altered MHC‐I expression [69]. Therefore, CRLM cells evade immune elimination by simultaneously impairing adaptive immune recognition and engaging inhibitory pathways within innate immune surveillance.

Overall, impaired immune visibility represents one of the major mechanisms underlying effector failure in CRLM. Tumor cells reduce antigen presentation and IFN‐γ responsiveness, whereas the liver metastatic niche reinforces inhibitory checkpoint signaling and weakens the local immune‐organizing circuits needed for sustained antigen‐dependent immunity.

### 3.2 Impaired Cytotoxic Function and Effector Cell Dysfunction

Even after tumor recognition, effective antitumor immunity ultimately relies on the ability of effector cells to execute cytotoxic responses. CD8^+^ T cells exhibit diverse killing mechanisms. They eliminate cancer cells primarily by releasing cytotoxic granules, including perforin and granzyme B (GZMB), which induce apoptosis by activating caspase pathways or interacting with proapoptotic proteins such as BH3‐interacting domain death agonists [70, 71, 72]. Moreover, CD8^+^ T cells express the Fas ligand, which binds to the Fas receptor on cancer cells, thereby initiating the extrinsic apoptotic signaling cascade [73]. Importantly, cytotoxicity is not restricted to apoptosis, as CD8^+^T cells can also induc+e nonapoptotic programmed cell death, most notably ferroptosis, thereby broadening the potential tumoricidal outcomes [74]. IFN‐γ produced by CD8^+^ T cells further amplifies antitumor immunity by increasing macrophage tumoricidal activity [75, 76], promoting inflammatory polarization and destabilizing Treg‐mediated suppression [76, 77]. The mechanism by which CD8^+^T cells kill CRC is summarized in Figure 2.

**FIGURE 2:**
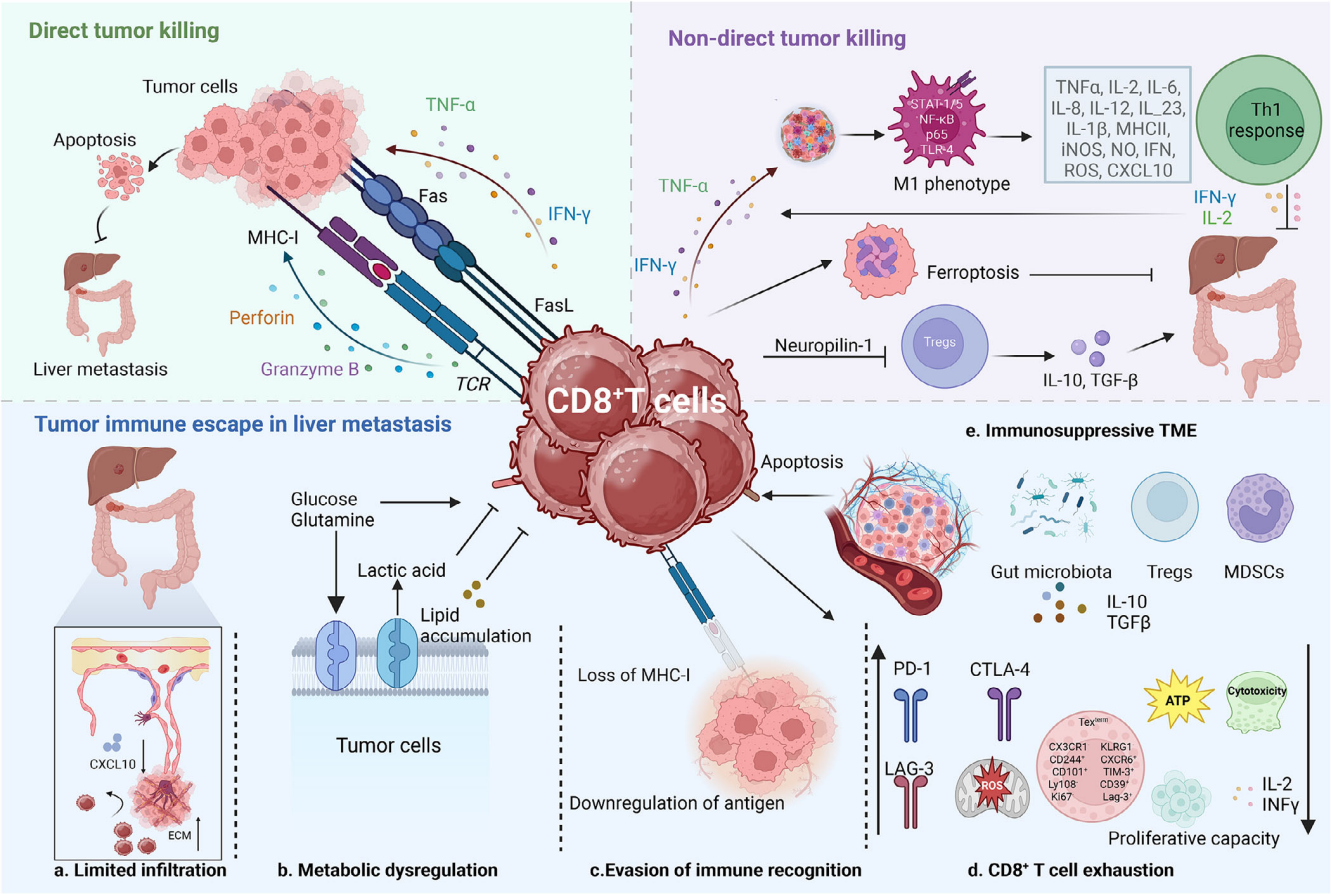
CD8^+^ T cell‐mediated immune responses in colorectal cancer liver metastasis. CD8^+^ T cells recognize tumor cells via MHC‐I‐presented tumor antigens and induce cytotoxicity through perforin and granzyme B release, Fas/FasL signaling, and IFN‐γ and TNF‐α secretion. These mechanisms enable direct lysis of metastatic colorectal cancer cells in the liver. In addition to their direct cytotoxic effects, CD8^+^ T cells can indirectly exert antitumor effects by modulating other immune cell populations within the tumor microenvironment(TME), including macrophages and Tregs, and by inducing alternative forms of cell death. However, multiple tumor escape strategies impair CD8^+^ T‐cell infiltration, activation, and persistence. Abnormal vasculature and downregulation of the expression of chemokines (e.g., CXCL9/CXCL10) hinder T‐cell trafficking into the TME. CRC cells in the liver often downregulate MHC‐I expression or alter antigen presentation pathways to evade immune detection. Within the TME, nutrient competition, hypoxia, and lactate accumulation result in metabolic dysfunction of CD8^+^ T cells, limiting their proliferation and effector function. Chronic antigen stimulation leads to exhaustion, which is marked by the upregulation of the expression of inhibitory receptors such as PD‐1, TIM‐3, and LAG‐3. Furthermore, immunosuppressive cells and cytokines—including regulatory T cells, TGF‐β, and IL‐10—create a tolerogenic liver environment that inhibits CD8^+^ T‐cell activity.

However, in established CRLM, this cytotoxic function is progressively impaired. Owing to persistent exposure to tumor antigens, continuous stimulation by immunosuppressive cytokines, metabolic stress, and inhibitory cell–cell interactions, CD8^+^ T cells gradually enter dysfunctional states. Importantly, immune dysfunction in patients with CRLM is not simply characterized by reduced immune cell infiltration; instead, infiltrating cytotoxic lymphocytes frequently undergo functional alterations that limit their ability to eliminate tumor cells. Single‐cell transcriptomic studies comparing primary CRC and CRLM have demonstrated substantial remodeling of T‐cell states during metastatic progression, with enrichment of exhausted CD8^+^ T‐cell populations characterized by increased inhibitory receptor expression, reduced effector molecule production, and impaired proliferative potential [78, 79]. Thus, CRLM progression involves functional reprogramming of infiltrating immune cells, rather than simply limiting immune cell recruitment.

The liver is enriched in NK cells, including conventional NK cells and liver‐resident NK (Lr‐NK) cells. Lr‐NK cells possess enhanced cytotoxic potential because of increased intracellular levels of lytic granules [80]. NK cells represent another major cytotoxic population and are particularly important in the liver [81, 82]. NK cells eliminate CRC cells through perforin and granzyme release, as well as antibody‐dependent cellular cytotoxicity (ADCC) [83, 84, 85]. In addition to direct cytotoxicity, NK cells shape the immune landscape of CRC by promoting CD8^+^ and CD4^+^ T‐cell activation, limiting Treg expansion, enhancing IFN‐γ production, and facilitating dendritic cell (DC) cross‐presentation [86, 87]. Therefore, NK‐cell dysfunction not only reduces innate cytotoxicity but also impairs their ability to support adaptive antitumor immune responses.

Single‐cell RNA sequencing and spatial transcriptomic analyses have revealed heterogeneity among NK cell subsets. In CRC and CRLM, the number of activated NK cells is diminished, while GZMK^+^ resting NK cells accumulate. These cells exhibit tumor‐promoting characteristics through intercellular interactions, promoting the dissociation of stem cell factor on the tumor cells membrane [88]. Additionally, in the liver metastatic niche, HSCs secrete CXCL12, which drives NK cells toward resting states, reducing their antitumor efficacy [89]. The suppressive cytokine milieu impairs NK cell metabolism and cytotoxicity. The suppressive immune cells present in the TME, such as Tregs, secrete many cytokines, especially TGF‐β [90, 91, 92], which blocks IL‐15‐induced mTOR activation and suppresses NK cell metabolic activity, proliferation, and receptor expression, which significantly reduces NK cell cytotoxicity [93]. TGF‐β also drives the transformation of circulating cytotoxic NK cells into less effective ILC1‐like phenotypes, further impairing their function [94].

Lr‐NK cells play an important role in immune‐mediated clearance of CRLM. The hypoxic and glycolytic environment of liver metastases results in lactic acid accumulation, resulting in intracellular acidification and apoptosis of Lr‐NK cells [95], exacerbating CRLM. Additionally, the transcription factor RORα is crucial for Lr‐NK cell maintenance and function; conditional knockout of RORα in mice leads to accelerated CRLM progression [96, 97]. Therapeutically, cytokines such as IL‐15 and IL‐2 may represent potential strategies to enhance NK‐cell activity and antimetastatic immunity [89, 98].

Collectively, CRC cells and the metastatic liver microenvironment impair CD8^+^ T cell and NK cell functions through persistent antigen stimulation, inhibitory signaling, metabolic restriction, and altered cellular communication. Thus, in CRLM, infiltrating immune cells undergo progressive functional deterioration, resulting in reduced killing capacity and impaired coordination of antitumor responses.

### 3.3 Disrupted TLS Organization and Impaired Local Antitumor Immunity

The third mechanism underlying effector dysfunction is the failure to sustain organized local immunity. Effective local immunity depends not only on cytotoxic lymphocyte infiltration but also on local immune structures that coordinate antigen presentation, B‐cell activation, T‐cell priming, chemokine‐guided recruitment, and effector cell persistence. In this context, B cells and TLSs represent important components of the local immune organization.

Traditionally, the antitumor effects of B cells were mediated primarily through antibody production, thereby facilitating ADCC and antibody‐dependent cellular phagocytosis (ADCP) [99, 100]. In the context of liver metastases, KCs can mediate ADCP and eliminate tumor cells [101], which is consistent with clinical associations between increased B‐cell density and improved CRC outcomes [102].

However, CRLM frequently imposes a distinct constraint on B‐cell immunity. B‐cell density is reduced relative to that in primary lesions, and residual B‐cell populations can display attenuated responsiveness to immunotherapy and other treatments []. Moreover, within the metastatic liver environment, activated memory B cells frequently acquire immunosuppressive phenotypes such as regulatory B cells, which contribute to immune evasion and the establishment of a tumor‐promoting niche in the liver [104]. Mechanistically, this duality is reinforced by evidence that B cells can promote tumor growth and dissemination through immunosuppressive cytokines such as IL‐10 [105, 106]. However, the broader functional and surface phenotypic heterogeneity of tumor‐infiltrating B cells remains difficult to fully define because of limited discriminative markers [107]. Single‐cell transcriptomic profiling has begun to clarify these dynamics in CRC, revealing depletion of activated B‐cell states in CRLM and implicating impaired migratory and functional circuits. Specifically, the SDF1–CXCR4 axis, which is crucial for B‐cell trafficking and activity, is suppressed by tumor‐intrinsic Wnt and TGF‐β signaling, potentially limiting B‐cell recruitment and antimetastatic functions. In addition, scRNA‐seq has revealed a novel B‐cell subset, termed the immature plasma cell population alpha, which is derived from activated B cells and strongly correlates with metastatic potential [108].

TLSs provide organized immune niches through which B‐cell‐associated immunity supports sustained effector responses [109]. Mature TLSs contain organized B‐cell follicles, T‐cell zones, DCs, and high endothelial venule‐like structures, these structures facilitate local antigen presentation and lymphocyte priming [110]. Clinically, TLS density is correlated with improved outcomes in patients with CRLM [111, 112, 113], but these associations are influenced by TLS localization, maturity, and immune composition [113, 114, 115, 116]. TLSs located at the invasive margin of CRLM are associated with favorable prognosis, and broader CRC studies indicate that TLS‐rich tumors display more active antitumor immune states [117]. These findings support the view that TLSs function as active immune niches.

Mechanistically, CXCL13 signaling links tumor reactive T cells, B cells, and TLS formation. CXCL13 recruits CXCR5^+^ B cells and contributes to lymphoid follicle organization, whereas CCL19 and CCL21 support T‐cell and DC recruitment [102, 118]. Activated B cells enhance CXCL13 and GZMB secretion by CD103^+^CD8^+^ tissue‐resident memory T cells and thereby strengthening local tumor control [119]. CXCL13 was initially characterized as a molecular marker of CD8^+^ T‐cell exhaustion [120]. In the TME, exhausted T cells are heterogeneous and can retain tumor reactivity with variable cytotoxic potential [121]. This heterogeneity indicates that CXCL13^+^ T cells are not necessarily terminally dysfunctional and may retain tumor‐reactive potential. Relatedly, CXCL13 has emerged as a robust marker of tumor‐specific T cells in multiple malignancies [122, 123].

Thus, effector dysfunction in CRLM represents a process involving multiple mechanisms. Tumor cells first reduce immune visibility through impaired antigen presentation and IFN‐γ responsiveness. Cytotoxic CD8^+^ T cells and NK cells are then restrained by checkpoint signaling, suppressive cytokines, stromal programs, and metabolic stress. Defective TLS organization prevents sustained local immune activation and effector persistence.

### 3.4 Spatial Immune Exclusion and Migratory Defects

A major limitation of CRLM is that effective immunity is often constrained at the level of immune cell delivery and positioning. In line with this observation, high CD3^+^ lymphocytes density at the tumor front in liver metastases is associated with recurrence risk and overall survival (OS) [124]. In CRLM, ineffective antitumor immunity often reflects not only an absolute deficiency of immune effector cells but also a failure of these cells to access, traverse, and become productively positioned within metastatic lesions. From a trafficking perspective, the entry of circulating T cells into tumor tissue requires a coordinated sequence of events, including adequate vascular perfusion, endothelial tethering, and firm adhesion, mediated by selectins and integrin ligands such as intercellular adhesion molecules (ICAMs) and vascular cell adhesion molecules, transendothelial migration, and subsequent movement along chemokine gradients within the tissue [125]. In CRLM, these critical steps are frequently impaired by endothelial dysfunction, culminating in the “vascular–immune barrier” that restricts effective immune cell entry into the tumor core.

Abnormal tumor vasculature is characterized by structural irregularities, including tortuosity and leakiness, together with inadequate perfusion and local hypoxia [126]. These abnormalities create a hostile TME that hinders immune cell trafficking. A key concept underpinning vascular restriction is that tumor endothelial cells can adopt a state of reduced inflammatory responsiveness, often referred to as endothelial anergy, in which the endothelial compartment becomes less capable of supporting lymphocyte arrest and transmigration [125]. These critical steps are frequently impaired by endothelial dysfunction, culminating in the “vascular–immune barrier,” which restricts effective immune cell entry into the tumor core in CRLM.

In CRLM, the tumor growth pattern, specifically the histopathological growth pattern (HGP) at the tumor‒liver interface, further modulates the vascular architecture and immune accessibility. CRLM exhibits distinct HGPs, including desmoplastic, pushing, and replacement patterns [126]. Desmoplastic and pushing HGPs rely on angiogenesis for tumor vascularization, whereas replacement HGPs involve tumor cells coopting the existing liver vasculature, a process known as vessel cooption. This distinction is critical, as angiogenic blood vessels, while abnormal, can still be normalized, leading to improved immune cell access. In contrast, coopted vessels are quiescent and less likely to support immune cell recruitment and migration, thereby contributing to a more pronounced immune‐excluded phenotype [126, 127]. Zhang et al. reported that angiogenesis‐driven HGPs were more susceptible to vascular normalization therapies, whereas vessel cooption‐related HGPs maintained immune exclusion, particularly following anti‐vascular endothelial growth factor (VEGF) therapy. Notably, anti‐VEGF treatment may promote vessel cooption, thereby reshaping the immune microenvironment. In CRLM models, bevacizumab shifted tumor growth toward replacement HGPs, promoted vessel cooption, and aggravated immune exclusion. Mechanistically, this treatment induced HSCs to upregulate FAPα expression, which, through FGF pathways, contributed to CXCL5 secretion, activating the CXCR2 axis and enhancing MDSCs infiltration. This inflammatory cascade promoted EMT in tumor cells and reinforced the vessel cooption phenotype [128].

Even after crossing the vascular barrier, immune cells encounter another major obstacle in CRLM: a fibrotic ECM barrier driven by HSC‐derived CAFs, which restricts deep tumor infiltration. The liver microenvironment induces immune exclusion through metastatic seeding and stromal remodeling. During metastatic colonization, CTCs interact with resident stromal and endothelial cells, altering their normal functions: CTCs interact with LSECs, driving a phenotypic shift from tumor‐suppressive functions toward facilitating metastatic colonization [129]. Under pathological conditions, LSECs undergo capillarization, thereby losing their protective properties and promoting angiogenesis and vasoconstriction [130]. In response to CTC exposure, LSECs secrete tumor necrosis factor‐alpha (TNF‐α), which upregulates matrix metalloproteinase‐9 (MMP9) expression and promotes the formation of intracellular gaps that enable tumor cell extravasation into the space of Disse and hindering CD8^+^ T‐cell infiltration [131]. Metastatic CRC cells establish a microenvironment characterized by HSC activation and the recruitment of immunosuppressive cells. In CRLM, HSCs represent a major source of collagen‐producing activated stromal cells and exhibit significant heterogeneity. Activated HSCs and macrophage‐derived granulin initiate fibrotic processes, including ECM production and angiogenesis [132, 133]. The resulting stiffened ECM and suppressive milieu jointly enforce immune exclusion and support CRLM outgrowth [134]. These dense ECM‐rich regions not only impede immune cell migration but also contribute to immune suppression. Spatial and in situ sequencing studies have shown that the fibrotic rim is biologically zoned, with the outer layers exhibiting generalized liver repair responses and the inner layers expressing more tumor‐supportive programs, further enhancing ECM deposition and tumor progression [135]. Collectively, these findings suggest that the fibrotic rim serves as both a physical and functional barrier that limits T‐cell penetration while fostering an immunosuppressive microenvironment.

Moreover, ECM remodeling in CRLM is not only structural but also profoundly instructive at the signaling level. The FAPα^+^HSC–CXCL5–CXCR2 axis promotes MDSC infiltration in parallel with impaired CD8^+^T‐cell entry, resulting in a spatial configuration in which suppressive cells accumulate where effector T cells are least able to penetrate [128]. These convergent mechanisms help explain a clinically relevant phenotype: compared with primary CRC and pulmonary metastases, patients with CRLM typically exhibit markedly reduced CD8^+^ T‐cell infiltration, thereby contributing to poor immune checkpoint inhibitor (ICI) responsiveness in the context of liver metastasis [136].

Thus, even when effector immunity is present systemically, CRLM establishes an environment that limits immune access.

### 3.5 Metabolic Reprogramming and Immune Metabolic Competition in CRLM

In addition to physical exclusion caused by the abnormal vasculature and fibrotic ECM, metabolic changes within the liver metastatic microenvironment further impair antitumor immunity. Tumor cells in CRLM compete with immune cells for essential nutrients, while increased glycolytic activity and lactate accumulation impair the function of infiltrating lymphocytes. Moreover, changes in lipid metabolism and BA signaling reshape the function of immune and stromal cells, promoting the establishment of an immunosuppressive microenvironment. These metabolic changes are interconnected and allow CRC cells to maintain nutrient and metabolic advantages while simultaneously weakening immune surveillance.

#### 3.5.1 Nutrient Competition Between Tumor Cells and Immune Cells in CRLM

Within the metabolic microenvironment of CRLM, competitive metabolic interactions among immune cells constitute another critical dimension of tumor immune evasion. The competition between tumor cells and infiltrating lymphocytes for limited nutrients is particularly pronounced: by upregulating both glycolysis and mitochondrial metabolism, tumor cells outcompete CD8^+^ T cells in the uptake of key substrates, including glucose, glutamine, and fatty acids, resulting in constrained effector T‐cell metabolism and impaired function [137]. Amino acid metabolic competition exacerbates this asymmetry. Tumor cells and MDSCs highly express Arginase‐1, which competitively depletes microenvironmental L‐arginine, leading to CD3ζ chain downregulation and impaired TCR signaling in T cells, thereby restricting T‐cell proliferation and effector function [138, 139]. Arginine metabolites can also promote tumor cell proliferation and M2‐like macrophages polarization, exacerbating immunosuppression [140]. In addition, indoleamine 2,3‐dioxygenase 1, which is expressed by tumor cells and immunosuppressive cells, catalyzes the conversion of tryptophan into kynurenine. Tryptophan depletion activates the GCN2 stress kinase pathway in T cells, precipitating functional inactivation; in turn, accumulated kynurenine activates the aryl hydrocarbon receptor transcriptional program to induce Treg differentiation while suppressing effector T‐cell function [141]. Furthermore, MDSCs efficiently sequester extracellular cystine through SLC7A11 to sustain their own glutathione synthesis and redox homeostasis while simultaneously depriving T cells of cysteine, an amino acid essential for T‐cell activation and proliferation [142]. Notably, immune cell competition extends beyond small‐molecule nutrients to encompass metabolic deprivation at the cytokine level. Through high CD25 expression, Tregs competitively consume microenvironmental IL‐2 via their high‐affinity trimeric receptor, thereby depriving effector T cells of the key growth factor needed for their survival and proliferation [143]. In the context of the distinctive metabolic background of CRLM, the combined effect of elevated Treg proportions and systemic local IL‐2 sequestration inhibit the metabolic activity of CD8^+^ T cells.

This metabolic plasticity is not a passive adaptation but a key determinant of immune exclusion. As metastatic lesions expand, tumor metabolic demand and disordered perfusion result in the generation of spatially heterogeneous microdomains of nutrient depletion and metabolite accumulation [96, 144, 145]. These conditions preferentially impair effector lymphocytes, which require high metabolic flexibility to maintain proliferation and cytotoxicity. In this context, immune infiltration is not only spatially restricted but also metabolically unsustainable once immune cells enter the tumor bed.

#### 3.5.2 Lactate Accumulation Impairs Immune Function and Alters Signaling Pathways

In addition to nutrient competition, highly glycolytic tumors convert pyruvate to lactate, which is exported via monocarboxylate transporters, leading to lactate accumulation and extracellular acidification. Lactate also functions as an immunoregulatory molecule that influences lymphoid and myeloid cell functions.

At the level of cellular metabolism, tumor‐derived lactate can rewire T‐cell metabolism and function, contributing to impaired effector programs in acidic tumor contexts. Furthermore, lactate stress is linked to altered TGF‐β signaling, reduced cytokine production, and reduced cytolytic activity in CD8^+^ T cells [146]. This glycolytic shift can occur even under oxygen‐rich conditions [147] and is driven by hypoxia‐inducible factor‐1α activated through β‐catenin and ROS/PI3K/Akt signaling [148]. In addition to immediate suppression, enforced glycolytic metabolism can impair long‐term memory CD8^+^ T‐cell formation [149], whereas mitochondrial insufficiency can precipitate metabolic reprogramming, which promotes functional exhaustion [150]. In addition to lymphocytes, lactate can remodel myeloid compartments, which are particularly relevant in liver metastases, as macrophage populations are abundant and intrinsically tolerogenic. The tumor‐resident microbiota increases lactate production in CRLM and promotes M2 macrophage polarization through RIG‐I lactylation, thereby suppressing NF‐κB recruitment to the Nlrp3 promoter and reducing inflammatory transcriptional outputs [146].

#### 3.5.3 BA Metabolism and Immune Regulation in CRLM

In CRLM, metabolic stress is further influenced by the unique metabolic environment of the liver. Hepatic metabolism provides organ specific signals that influence immune tolerance and tumor progression. In particular, BAs act as liver‐derived metabolic signals that regulate immune responses.

Secondary BAs, influenced by the gut–liver axis, can regulate the metastatic microenvironment in CRLM. Lithocholic acid can signal through TGR5 in hepatic stromal compartments to induce chemokine programs that recruit suppressive CCR1^+^ MDSCs, contributing to an immunosuppressive metastatic microenvironment [151]. Similarly, BAs increased the migration capability of CRC cells and promoted CRLM in a mouse model by activating HSCs, increasing fibroblast activation, and expanding myeloid‐derived immune cell populations [152]. Clinically relevant disturbances in BA homeostasis can alter CD8^+^ T‐cell activation and cytotoxicity and promote CRLM progression by increasing CXCL5/CXCR2‐mediated neutrophil recruitment [55, 153]. These findings indicate that BA metabolism actively connects hepatic metabolic changes with immune suppression in CRLM.

#### 3.5.4 Lipid Metabolism and Ferroptosis‐associated Immune Regulation in CRLM

In addition to glucose and BA metabolism, lipid metabolism emerges as a dominant liver‐metastasis–selective axis that coordinates both tumor fitness and immune suppression. Multiomic analyses of CRLM demonstrate extensive lipid accumulation and dysregulated lipid metabolic pathways. Multiomic profiling of clinical CRLM has revealed broad dysregulation and accumulation of lipid metabolites; functionally, lipid deposition can promote metastatic capacity through an epitranscriptomic–metabolic mechanism in which YTHDF3 enhances m6A‐dependent degradation of PPARα, thereby disrupting lipid catabolic control [154].

Mechanistically, tumor cells can satisfy their lipid demand through both enhanced de novo fatty acid synthesis (FAS) and increased fatty acid uptake. CRC cells have been reported to downregulate succinate dehydrogenase, activating the PI3K/AKT signaling axis to promote lipid accumulation and reduce the rate of fatty acid oxidation, thereby facilitating CRC cells growth and contributing to the development of CRLM [155]. In the liver, excessive cholesterol accumulation can lead to the formation of lipid droplets, impair T cell function, and provide a favorable environment for CRC cells, thereby promoting CRLM [156]. Additional tumor intrinsic alterations, such as PTPRO silencing, enhance ACC1‐mediated lipogenesis and further promote metastatic competence [157]. Consistently, in the context of nonalcoholic fatty liver disease, altered lipid availability can promote CRLM via lipid handling programs that enhance EGFR palmitoylation, linking hepatic metabolic disease to metastatic competence [158]. Finally, lipid regulatory circuits are directly co‐opted to reshape liver resident immune cell heterogeneity and functional states, reinforcing immune suppression. Macrophages exhibit particularly active lipid metabolism, and recent spatial and single cell analyses suggest that lipid‐metabolic programs are elevated in macrophage populations during CRLM progression and are associated with distinct macrophage states [159]. Metabolic reprogramming in macrophages increases utilization of triglycerides and free fatty acids, promotes an immunosuppressive TME, and contributes to CD8^+^T cell exhaustion in association with M2 like features [160]. In liver metastases, CRC cells enhance exhaustion through upregulation of glycerol‐3‐phosphate acyltransferase 3, which drives lipid droplet accumulation and suppresses immunogenic cell death, accelerating both immune evasion and malignant progression [161].

As the central hub for systemic iron homeostasis and lipid metabolism, the liver harbors an intrinsic ferroptotic pressure field characterized by high iron content and a polyunsaturated fatty acid‐enriched microenvironment. Disseminated CRC cells that seed the liver are confronted with abundant free Fe^2^^+^ released from KC‐mediated phagocytosis of senescent erythrocytes, which continuously drives membrane phospholipid peroxidation through the Fenton reaction, thereby rendering the liver the most stringent ferroptotic barrier along the metastatic cascade. Guo et al. proposed the concept of a “ferroptosis filter,” positing that ferroptosis imposes a selective barrier at each step of metastasis, such that only clones equipped with robust antioxidant capacity can complete intrahepatic colonization [162]. This conceptual framework directly links the unique hepatic biochemical environment to the selection of metastatic clones. At the molecular level, CRC liver‐metastatic cells adapt to oxidative stress by upregulating arylacetamide deacetylase, which activates the NRF2 transcriptional program and induces the expression of SLC7A11 (the catalytic subunit of System Xc−). This adaptive reprogramming enhances cystine uptake and glutathione synthesis, thereby restraining lipid peroxidation and facilitating tumor growth in the liver [163]. Furthermore, ferritinophagy serves as a critical regulatory node in hepatic iron‐overload‐driven ferroptosis. NCOA4‐mediated selective autophagic degradation of ferritin has been identified as a central mechanism precipitating ferroptosis under iron‐overload conditions; consequently, successfully colonized CRC cells must concomitantly suppress ferritinophagy to prevent the uncontrolled expansion of the labile iron pool [164]. Collectively, the high‐ferroptotic‐pressure hepatic microenvironment functions both as a selective barrier against disseminated tumor cells and as a potent driver of ferroptosis‐resistant clones—whose ferroptosis defense programs, in turn, set the stage for subsequent immune evasion. It has been reported that fatty acids accumulating in the TME are taken up by CD8 T cells through the scavenger receptor CD36, whereupon they directly trigger T cell ferroptosis by amplifying lipid peroxidation and intracellular iron accumulation, resulting in markedly diminished IFN‐γ and TNF‐α secretion and severely compromised cytotoxic function [165]. In the context of liver metastases, the liver itself is the central organ for cholesterol and lipoprotein metabolism, and the concentration of oxidized lipids within the hepatic TME far exceeds that in other organs—endowing the CD36–CD8 T cell ferroptosis axis with particularly salient pathological significance in CRLM. In striking contrast, immunosuppressive cell populations have acquired a “selective survival advantage” over CD8 T cells amid the ferroptosis‐pressured milieu of CRLM, through multiple layers of defense programs [166]. Tregs safeguard themselves against lipid peroxidation and ferroptosis by upregulating GPX4 [167]. MDSCs rely on a four‐tier ferroptosis defense system: N‐acylsphingosine amidohydrolase 2 suppresses the p53–Hmox1 axis to curtail ROS production; fatty acid transporter protein 2 channels arachidonic acid toward PGE2 synthesis, simultaneously reducing their own lipid peroxidation burden while releasing PGE2 to suppress T cells; xCT/SLC7A11‐mediated cystine competition depletes microenvironmental cysteine; and the aconitate decarboxylase 1‐itaconate–nuclear factor erythroid 2‐related factor 2 pathway provides an additional layer of antioxidant protection [168]. Together, these coordinated mechanisms enable MDSCs to selectively persist under the extreme oxidative stress conditions of the TME.

In aggregate, resistance in CRLM can be understood as a layered process: restricted infiltration, followed by metabolic suppression, immune escape, and exhaustion. These lesion‐level mechanisms are embedded within a broader immunosuppressive ecosystem defined by liver tolerance, suppressive cell networks, cytokines, and distal regulators.

### 3.6 Immunosuppressive Immune Networks in CRLM

#### 3.6.1 Liver Resident Cells Drive Immunosuppressive Remodeling in CRLM

The intrinsic tolerogenic properties of the liver promote metastatic immune escape [169]; CRC cells colonize; they further strengthen this suppressive state through interactions with liver‐resident cells and recruitment of immunosuppressive populations.

HSC activation represents a key step in the remodeling of the hepatic metastatic microenvironment. Under physiological conditions, HSCs retain quiescent vitamin A‐storing cells; however, during metastatic colonization, tumor‐derived EVs and cytokines drive HSCs toward activated inflammatory and fibrogenic phenotypes [170]. Importantly, in CRLM, activated HSCs function beyond ECM producing cells and acquire immunoregulatory properties. Activated HSCs produce CCL2 and CXCL12, which promote the recruitment of CCR2^+^ monocytes and facilitate their differentiation into immunosuppressive macrophages [171]. Moreover, HSC‐derived IL‐6, TGF‐β, and GM‐CSF reinforce myeloid polarization and suppress effective antitumor immune responses, connecting stromal remodeling with immune escape [172].

KCs are also integrated into this immunosuppressive network. Under physiological conditions, KCs act as resident hepatic sentinels, whereas during metastatic colonization they undergo functional reprogramming toward an IL‐10/TGF‐β‐rich state with reduced antigen‐presenting capacity [18]. This phenotypic shift is driven by continuous crosstalk with monocytes and HSC‐derived mediators, which together reinforce local immunosuppression. Reprogrammed KCs further promote the recruitment of circulating CCR2^+^ monocytes while impairing effective CD8^+^ T cell priming, thereby favoring the establishment of a tolerogenic hepatic microenvironment. Consistent with this notion, bulk RNA sequencing revealed KCs were recruited and polarized to M2 phenotype though CXCL16/PI3K/AKT/FOXO3a pathway in CRLM [173].

LSECs contribute to a distinct but highly integrated layer of immune suppression by reshaping antigen presentation into a tolerogenic form. In CRLM, LSECs exhibit reduced costimulatory signaling while maintaining antigen uptake capacity, thereby promoting T‐cell anergy rather than activation. Recent vascular immunology studies have demonstrated that LSECs also act as immune gatekeepers, controlling leukocyte trafficking specificity and selectively facilitating the infiltration of immunosuppressive myeloid subsets while restricting cytotoxic T‐cell entry [174]. This endothelial reprogramming is reinforced by TGF‐β and VEGF signaling, linking vascular remodeling directly to the immune exclusion architecture. LSECs exhibit immunosuppressive features even at rest and can inadvertently protect metastatic cells from immune surveillance [175].

Hepatocytes can further shape the metastatic environment through the release of soluble mediators that support tumor cell survival and suppress antitumor immunity. For example, hepatocyte‐derived FGL1 has been shown to limit T‐cell infiltration [176]. Together, these liver resident cell populations establish a microenvironment in which fibrosis, vascular remodeling, defective antigen presentation, and myeloid recruitment reinforce one another to promote immune suppression and metastatic colonization. The effects of hepatic resident cells on CRLM are summarized in Table 2.

**TABLE 2:** Liver‐resident cell alterations supporting colorectal cancer liver metastasis.

Liver‐specific cells	Molecular signaling factors	Effect on CRLM	References
Hepatocytes	Fibrinogen‐like‐protein‐1	Reduce T‐cell infiltration	[176]
	Extracellular creatine	Promote cancer cells to produce ATP	[177]
	CCL3	Regulate cancer cell invasion	[178]
	IL6/STAT3/SAA pathway	Promote the formation of NETs and the infiltration of MDSCs	[179]
LSECs	TNF‐α, MMP9, and ICAM1	Promote actin disintegration and the formation of intracellular gaps; facilitate cancer cells infiltration and proliferation	[131]
	Cell adhesion molecules	Induce suppressive TME and facilitate the cancer cell colonization	[21, 179, 180, 181]
KCs	CXCL5	Recruit MDSCs	[182]
	Galectin‐9–dectin‐2 axis	Inhibit phagocytosis of cancer cells	[183]
	Inflammatory cytokines	Promote cancer cells invasion and angiogenesis; induce a suppressive TME	[22]
HSCs	Cytokines	Regulate the growth and spread of cancer cells	[184, 185]
	Cytokines	Induce the differentiation of regulatory DCs and MDSC and T cells apoptosis	[186, 187]
	C5a and IL‐1β	Promote the recruitment and infiltration of neutrophils; mediate the formation of NETs	[186]

Abbreviations: ATP: adenosine triphosphate; NETs: neutrophil extracellular traps; MDSCs: myeloid‐derived suppressor cells; TME: tumor microenvironment; DCs: dendritic cells; LSECs: liver sinusoidal endothelial cells; KCs: Kupffer cells; HSCs: hepatic stellate cells; TNF‐α: tumor necrosis factor α; MMP9: matrix metalloproteinase‐9; ICAM1: intercellular adhesion molecule 1.

#### 3.6.2 Myeloid Cell Recruitment and Treg Expansion Sustain Immunosuppressive Tolerance

Recruited myeloid populations further reinforce local immunosuppression and contribute to the maintenance of a tolerogenic metastatic microenvironment. Among these cells, MDSCs are early and central effectors. Their expansion in CRLM has been associated with pathways such as S1PR1–STAT3 signaling, but their role extends beyond numerical increase. MDSCs suppress autologous T‐cell proliferation, contribute to PMN formation, and influence metastatic progression [188]. Notably, monocytic MDSCs promote metastatic reactivation through CCL7 secretion and CCR2–JAK/STAT3 activation in dormant tumor cells [189]. Importantly, MDSCs also function within a broader immunosuppressive network involving TAM‐like macrophages, Tregs, and exhausted effector lymphocytes. Through Arginase‐1, iNOS, ROS, IL‐10, TGF‐β, and PD‐L1, MDSCs impair TCR signaling, deplete local nutrients such as L‐arginine, and promote high dysfunctional T‐cell states. These factors also promote Treg differentiation and maintenance. In SMAD4‐deficient CRC, this loop can be intensified by the recruitment of CCR1^+^ granulocytic MDSCs through the CCL15/CCR1 axis, with TGF‐β‐dependent suppression of cytotoxic lymphocytes [190].

Within this ecosystem, Tregs operate as both products and amplifiers of suppression. FOXP3^+^ Tregs suppress DC function by reducing CD80 and CD86 expression [191]. These cells can eliminate CD8^+^ T cells and NK cells through perforin dependent cytotoxicity [145, 192] and secrete immunosuppressive cytokines such as IL‐10 to inhibit CD8^+^ T‐cell infiltration and antitumor activity [193, 194]. Tregs also suppress effector T‐cell function through the CD39–CD73 adenosinergic pathway. Tregs hydrolyze extracellular ATP to produce adenosine, which binds to its receptors on effector T cells, thereby reducing their activity and further suppressing antitumor immune responses [195, 196]. The hepatic microenvironment may further favor Treg differentiation through TGF‐β produced by LSECs, HSCs, and KCs [25]. Consistent with this, increased Treg infiltration has been associated with greater metastatic potential and poorer prognosis [92, 197]. Single‐cell transcriptome analyses revealed that Tregs are the predominant T‐cell subtype in patients with CRLM [198].

Reciprocal interactions between Tregs and MDSCs may further sustain immune suppression. In CRLM, CTLA‐4^+^PD‐1^+^ICOS^+^ Tregs have been reported to modify MDSCs, promoting their migration to distant tumor sites and impairing cytotoxic T lymphocyte (CTL) responses through clonal anergy [199]. MDSCs, in turn, can enhance Treg activity. Beyond their immunosuppressive effects, Tregs may also facilitate metastatic progression by increasing TGF β signaling and promoting EMT [92]. Importantly, compared with that in primary CRC, TGF β in CRLM not only drives T‐cell exclusion but also prevents the acquisition of a Th1 effector phenotype [200]. Tregs also interact with fibroblasts and endothelial cells to promote angiogenesis through VEGF‐A secretion [96].

The CRLM immune microenvironment is also influenced by signals originating outside the liver, particularly the gut microbiota. The gut microbiota can modulate CD8^+^ T‐cell responses and metastatic progression [10, 201]. Specific bacterial species, such as Fusobacterium nucleatum, have been associated with the development of an immunosuppressive TME characterized by reduced CD8^+^ T‐cell infiltration [202]. Similarly, E. coli suppresses CD8^+^ T‐cell activation via mTOR signaling and enhances the immunosuppressive ability of Tregs by promoting PD‐1 expression, contributing to CRLM and thus underscoring the complex interplay between the gut microbiome and antitumor immune responses. These observations have prompted interest in microbiota‐directed interventions, including fecal microbiota transplantation, probiotics, and prebiotics, as potential strategies to improve responses to immune checkpoint blockade [203, 204]. The mechanism of the immunosuppressive microenvironment within liver metastases is shown in Figure 3.

**FIGURE 3:**
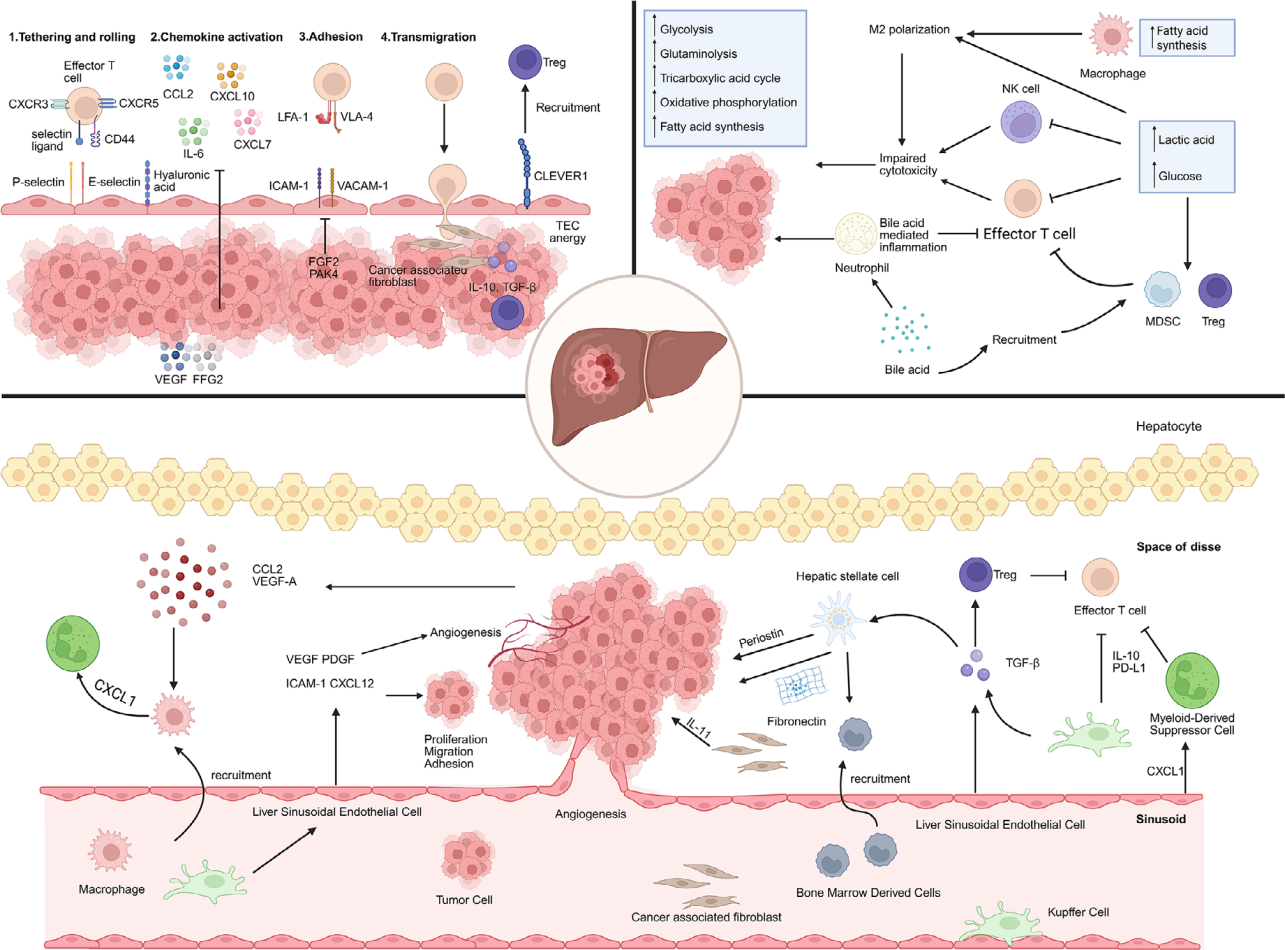
The cellular barrier, metabolic state, and hepatic niche shape immune suppression in colorectal cancer liver metastasis. First, cellular and vascular barriers restrict immune cell trafficking into metastatic lesions. Dysfunctional tumor endothelium, stromal remodeling and extracellular matrix deposition impair lymphocyte adhesion, transmigration, and intratumoral positioning. Second, metabolic reprogramming within metastatic tumors—including enhanced glycolysis, oxidative phosphorylation, lipid accumulation, and lactate production—results in nutrient competition and metabolic stress, which reduces NK and CD8^+^ T‐cell cytotoxicity while promoting immunosuppressive populations such as myeloid‐derived suppressor cells and regulatory T cells. Finally, the liver‐specific microenvironment reinforces immune tolerance. Hepatocytes, liver sinusoidal endothelial cells, Kupffer cells, and hepatic stellate cells coordinate cytokine signaling, stromal activation, and immune regulation, ultimately establishing a permissive niche that supports immune evasion and metastatic outgrowth.

### 3.7 Functional Diversity and Plasticity of Helper T Cells in CRLM

CD4^+^ helper T cells in CRLM display considerable functional diversity, and their effects depend strongly on the local context. Among CD4^+^ helper T‐cell subsets, Th1 cells show the most consistently antitumor activity in CRC and CRLM. Th1 cells can increase CRC immunogenicity by increasing the expression of HLA‐A and HLA‐DPB1 on tumor cells [76]. Their secretion of IFN‐γ and IL‐2 may also increase tumor sensitivity to CD8^+^ T‐cell‐mediated killing [205]. IFN‐γ promotes M1‐like macrophage polarization and DC activation and can enhance macrophage nitric oxide production as an effector mechanism [206, 207]. Clinically, elevated infiltration of Th1 cells has been associated with improved prognosis in CRC [60, 61, 197] and is generally associated with better responsiveness to ICI therapy [208]. In CRLM, however, Th1 responses are often attenuated: TGF‐β‐rich stromal and myeloid signals prevent effective Th1 polarization, and the broader suppressive network results in the dysfunction of both helper and cytotoxic T cells [209].

By contrast, Th2, Th17/Th22, and Th9 responses show greater context dependence, with both protumor and antitumor effects reported in CRC and CRLM. SPP1 signaling has been reported to interact with the CSF1 pathway and favor a shift from Th1‐ toward Th2‐dominant immunity in the TME, a change associated with metastatic progression [210]. In mouse models of CRC, Th2 cytokines lead to abnormal β‐catenin expression and tumor formation [211]. Th2 can favor metastatic progression in part through its effects on macrophages. IL‐4 and IL‐13 promote M2 macrophage polarization, which can in turn support Th2‐skewed immunity and favor immune escape [212]. Th2 responses, however, are not uniformly protumorigenic in CRC. Th2 cells enhance humoral immunity [213] and influence the polarization and activation of eosinophils. Eosinophils release cytotoxic mediators that may contribute to the suppression of CRC progression [214].

A similar context dependence is seen in Th17 and Th22 responses. In CRLM, IL‐17 and IL‐22 support metastatic progression by promoting cancer stemness, EMT [215], tumorigenic epithelial programs [216], and the invasion and metastasis of CRC [217].

In addition to IL‐17, IL‐22 plays a significant role in CRLM by inducing endothelial cell proliferation, chemotaxis, and neovascularization [218]. IL‐22 is also a major effector cytokine produced by Th22 cells. Both Th17 and Th22 populations accumulate in CRC, where IL‐17‐ and IL‐22‐associated responses may contribute to tumor development and progression [219]. IL‐22 levels are increased at CRLM, and IL‐22‐producing Th22 cells promote angiogenesis at established metastasis sites [220]. Notably, Th17 cells exhibit significant microenvironment dependent plasticity [221, 222]. Changes in the balance of Tregs and Th17 cells within the TME enable Th17 cells to undergo substantial developmental shifts and transform into Tregs [91]. In mouse models of CRC, the hepatic immune response is modulated by a reduction in Th17 cells and increased recruitment of Tregs, which promotes CRLM [223].

Although Th17 responses are often associated with tumor‐promoting effects, they can support antitumor immunity in some settings. They may recruit CTLs and DCs [145, 224] or even acquire Th1 like IFN‐γ‐producing features [221]. Their developmental relationship with Tregs is particularly relevant because a TGF‐β dominant metastatic niche can shift the Treg/Th17 balance toward immune tolerance.

Th9 responses also appear to be context dependent, although their specific role in CRLM remains less well defined. Experimental studies suggest that Th9 cells can maintain strong proliferative and cytolytic capacity and may be relatively resistant to exhaustion [225]. In preclinical models, Th9 cells have shown potent antitumor activity and, in some settings, have outperformed Th1 and Th17 cells [225, 226]. Th9 cells can support antitumor immunity by enhancing CTL responses [227] and promoting DC recruitment and antigen presentation [228]. Conversely, IL‐9 can promote the recruitment or activity of Tregs and mast cells [229] while Th9‐associated signaling has also been linked to EMT, angiogenesis, and tumor‐cell migration [230]. Taken together, available evidence suggests that Th9 function varies with the surrounding immune context, although its specific contribution to CRLM remains insufficiently defined.

### 3.8 Heterogeneity of the CRLM Immune Microenvironment

Although several immunosuppressive mechanisms are shared across CRLM, their extent and composition vary among patients, metastatic lesions, and stages of disease. The immune microenvironment of CRLM is highly heterogeneous and changes with tumor evolution, the hepatic tissue context, spatial organization, and therapeutic pressure. Recent single‐cell and spatial multiomics studies have demonstrated that metastatic immune landscapes continue to change during liver colonization and in response to treatment [78, 79, 103]. This heterogeneity has important clinical implications because immune features detected in the primary tumor or a single metastatic lesion may not accurately represent the immune status of the entire metastatic burden.

One major source of heterogeneity is the difference between primary CRC and matched liver metastases. Paired single‐cell studies have shown that the immune landscape of CRLM is not a simple copy of that of the primary tumor but is reshaped after tumor cells colonize the liver [79]. Some immune states are shared between primary and metastatic lesions and appear to be tumor associated, whereas others vary according to the tissue site. For example, exhausted CD8^+^ T cells and activated Tregs were classified as malignancy‐associated immune states, whereas NK cells and DC phenotypes were more dependent on the tissue environment [78]. Thus, metastatic tumor cells and the liver microenvironment jointly determine the final immune phenotype of CRLM. Compared with primary CRC, liver metastases tend to show stronger myeloid remodeling, including enrichment of SPP1^+^TAM and metabolically active MRC1^+^CCL18^+^ M2 macrophages [79]. Moreover, B cell, plasma cell, CD4^+^ T cells, and CD8^+^ T cells are reduced or functionally altered in liver metastases compared with those in primary tumors [231]. These differences caution against using the immune profile of the primary tumor as a direct surrogate for that of CRLM.

Immune heterogeneity is also evident within individual liver metastases. A single CRLM lesion contains multiple spatial compartments, including the tumor core, invasive margin, fibrotic interface, and surrounding liver parenchyma. These regions differ substantially in terms of the immune cell density, stromal composition, vascular structure, and cellular communication networks. Spatial transcriptomic analyses have demonstrated that immunosuppressive macrophages occupy distinct regions within CRLM. MRC1^+^CCL18^+^ macrophages preferentially localize within metastatic regions enriched for suppressive stromal programs, whereas SPP1^+^ macrophages interact closely with CAFs and form macrophage–fibroblast regions associated with reduced immune infiltration and metastatic growth [79, 232]. Thus, immune suppression in CRLM is determined not only by the presence or abundance of immune populations but by where they are located.

Further heterogeneity is seen between metastatic lesions within the same patient. In patients with multifocal CRLM, individual lesions can differ markedly in immune cell density, stromal organization, and cellular composition despite exposure to the same systemic environment. One lesion may exhibit immune infiltration and active immune surveillance, whereas another may display immune exclusion, stromal dominance, or myeloid enrichment. An evaluation of multiple resected CRLM lesions demonstrated that immune homogeneity among metastatic deposits was associated with favorable outcomes and provided prognostic information beyond the pathological response to preoperative chemotherapy [233]. Consequently, intermetastatic immune heterogeneity is not only a biological observation but also a clinically relevant prognostic feature.

## 4 The Management of CRLM

The management of CRLM should not be viewed as a selection among isolated treatment modalities but rather as a dynamic process in which local and systemic therapies are integrated according to resectability, tumor biology, molecular subtype, treatment response, and the presence of extrahepatic disease. At the initial diagnosis, patients should be evaluated by a multidisciplinary team, including hepatobiliary surgeons, medical oncologists, radiologists, interventional radiologists, radiation oncologists, and pathologists. The principal clinical objective is to determine whether all macroscopic disease can be eradicated immediately, after systemic downstaging, or only through long‐term disease control.

Accordingly, patients can be broadly classified into four groups: those with upfront resectable disease, those with initially unresectable but potentially convertible liver‐limited disease, those with unresectable oligometastatic or liver‐dominant disease, and those with extensive systemic dissemination. This classification is not fixed. Repeated multidisciplinary reassessment is essential because responses to chemotherapy, targeted therapy, or immunotherapy may result in initially unresectable disease transforming into a state amenable to resection or local ablation. The treatment options for CRLM are summarized in Figure 4.

**FIGURE 4:**
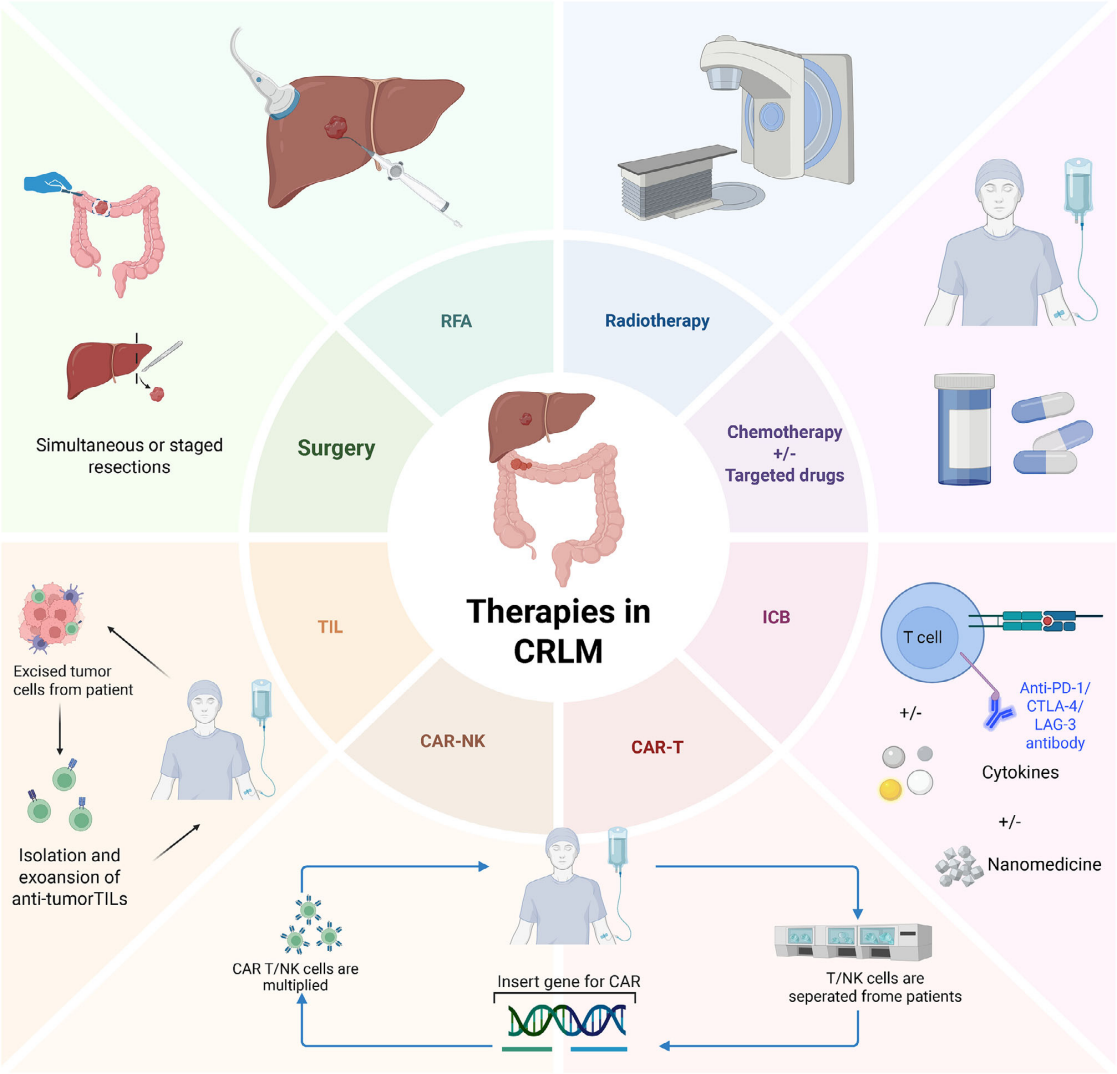
Current and emerging therapeutic strategies for colorectal liver metastases. Schematic overview of multidisciplinary treatment approaches for CRLM, including locoregional therapies, systemic treatments, and emerging immunotherapies. Surgical resection (simultaneous or staged hepatectomy and colorectal resection) remains the cornerstone for potentially curable disease, whereas radiofrequency ablation (RFA), radiotherapy, chemotherapy, and targeted agents provide complementary strategies for tumor control. Immune‐based approaches, including immune checkpoint blockade (ICB), tumor‐infiltrating lymphocyte (TIL) therapy, and CAR‐T and CAR‐NK cell therapies, represent promising next‐generation treatments. Novel delivery platforms, such as nanomedicine and cytokine‐based modulation, are being explored to enhance antitumor immune responses and therapeutic efficacy. RFA: radiofrequency ablation; ICB: immune checkpoint blockade; CAR‐T: chimeric antigen receptor T‐cell immunotherapy; CAR‐NK: chimeric antigen receptor NK cell immunotherapy; TIL: tumor‐infiltrating lymphocyte therapy.

### 4.1 Upfront Resectable CRLM: Surgery and Perioperative Systemic Therapy

Liver resection (LR) remains the principal potentially curative treatment for patients in whom all hepatic disease can be completely removed while preserving adequate liver function [234]. The choice between simultaneous and staged resection of the primary CRC and synchronous liver metastases depends on the complexity of the colorectal and hepatic procedures, the patient's physiological status, tumor burden, and institutional expertise [235, 236, 237]. Rather than favoring one strategy universally, surgical timing should be individualized through multidisciplinary discussion.

An important unresolved issue is whether patients with technically resectable CRLM should undergo immediate resection or receive perioperative chemotherapy. In the EORTC 40983 trial, compared with surgery alone, perioperative FOLFOX improved progression‐related outcomes but did not result in a statistically significant OS advantage after long‐term follow‐up [238]. This approach may be particularly useful in patients with a high metastatic burden, synchronous disease, an unfavorable disease‐free interval, elevated levels of tumor markers, or uncertain tumor biology because the response to systemic therapy can provide information regarding disease aggressiveness. Conversely, immediate surgery may be reasonable for patients with limited and clearly resectable disease when delaying local treatment is unlikely to provide additional benefits.

The type of perioperative systemic therapy is also important. The long‐term results of the New EPOC trial revealed that adding cetuximab to perioperative chemotherapy in patients with operable, KRAS wild‐type CRLM worsened clinical outcomes [239]. Thus, treatment selection must consider both the response rate and biological interactions among systemic treatment, tumor evolution, and surgical timing.

Although surgical trauma may transiently trigger systemic inflammation, promote tumor cell dissemination, and inadvertently exacerbate disease progression [240] and alterations in immune cell distribution [241], these mechanisms should not hinder potentially curative resection. Rather, they help explain postoperative recurrence and support the development of perioperative biomarkers and rational adjuvant strategies. While curative LR remains the gold standard for patients with resectable CRLM, recurrence rates remain high, with up to 70% of patients experiencing relapse, most often within the first 2 years postsurgery, but recurrence continues to occur within a decade, and surveillance and consideration of repeat local treatment are integral components of long‐term management [234].

### 4.2 Initially Unresectable but Potentially Convertible CRLM

For patients with liver‐limited disease that is not initially resectable, systemic therapy should be administered with the explicit objective of achieving sufficient tumor regression to permit complete local treatment. In this context, regimens capable of substantially shrinking the tumor and converting to resectability while minimizing chemotherapy‐associated hepatic injury should be prioritized [242].

The side of the primary tumor and molecular status should be considered during treatment selection. Patients with left‐sided, RAS/BRAF wild‐type tumors are best treated with chemotherapy and anti‐EGFR antibodies, while FOLFOX ± bevacizumab treatment is preferred for patients with RAS or BRAF mutations or right‐sided tumors [243]. Current guidelines support the use of doublet chemotherapy for most patients, whereas triplet chemotherapy may be considered in appropriate patients with initially unresectable disease when conversion to local treatment is a realistic goal [244]. Ongoing clinical trials are investigating novel combinations and sequencing strategies involving systemic and local therapies, aiming to optimize outcomes in this heterogeneous patient population (Table 3).

**TABLE 3:** Exploring targeted therapy in colorectal cancer liver metastasis: a review of ongoing clinical trials.

Target	Intervention	Combination	Phase	References
KRAS	BBO‐11818	Pembrolizumab, pembrolizumab ± cis/carboplatin + pemetrexed, or cetuximab	I	NCT06917079
	Hydroxychloroquine	Methotrexate, capecitabine + bevacizumab	II	NCT06949982
	JAB‐21822	Standard second‐line chemotherapy ± bevacizumab	II	NCT06838338
	ALTA3263	−	I	NCT06835569
	Isunakinra	PD‐1/PD‐L1 inhibitor	II	NCT06634875
	LY4066434	Cetuximab or Nab paclitaxel gemcitabine or oxaliplatin or leucovorin or irinotecan or 5‐fluorouracil or carboplatin or cisplatin or pemetrexed or pembrolizumab	I	NCT06607185
	BGB‐53038	Cetuximab	I	NCT06585488
	ERAS‐007	Palbociclib	I/II	NCT05039177
BRAF	Encorafenib	Cetuximab + bevacizumab	II	NCT06411600
	Tunlametinib	Vemurafenib	III	NCT06008119
	ZN‐c3	Encorafenib + cetuximab	I	NCT05743036
	Hydroxychloroquine	Encorafenib + cetuximab	II	NCT05576896
	HLX208	−	II	NCT05127759
	HLX208	Cetuximab	II	NCT04984369
	ERAS‐007	Encorafenib + cetuximab	I/II	NCT05039177
EGFR	Amivantamab	FOLFIRI	III	NCT06750094
	Amivantamab	mFOLFOX6 or FOLFIRI	III	NCT06662786
	Cetuximab/panitumumab	FOLFOX	III	NCT06226857
	Cetuximab/panitumumab	Irinotecan/FOLFIRI ± bevacizumab	II	NCT04587128
	Panitumumab	FOLFIRI	III	NCT06509126
	Panitumumab	Valproic acid + irinotecan	II	NCT06714357
VEGF	Bevacizumab	Emavusertib + FOLFOX	I	NCT06696768
	Bevacizumab	mFOLFOX6 + riluzole	I	NCT04761614
	Bevacizumab	TAS‐102 + irinotecan	II	NCT04109924
	Bevacizumab	mFOLFOX6 + atezolizumab	III	NCT02997228
	Fruquintinib	FOLFIRI	I/II	NCT05522738
	Fruquintinib	Chidamide + sintilimab	II	NCT06685276
	Fruquintinib	Cardonilizumab + SBRT	/	NCT06551207
	Fruquintinib	Camrelizumab + HAIC	II	NCT06423937
	Fruquintinib	Tegafur gimeracil oteracil	II	NCT06255379
	Ramucirumab	TAS102	III	NCT03520946
	Aflibercept	FolFIR	/	NCT04397601
HER2	Tucatinib	mFOLFOX6	III	NCT05253651

Abbreviations: FOLFIRI: folinic acid, fluorouracil and irinotecan; mFOLFOX6: (modified)5‐fluorouracil, leucovorin, and oxaliplatin; FOLFOX: 5‐fluorouracil, leucovorin, and oxaliplatin; SBRT: stereotactic body radiation therapy.

Data sources: https://clinicaltrials.gov/.

The CAIRO5 trial is particularly relevant because it prospectively evaluated induction strategies in patients with initially unresectable, liver‐only metastases and incorporated standardized reassessment by a central liver expert panel. Its clinical implication extends beyond regimen selection, highlighting that systemic therapy and surgical evaluation should proceed in parallel. Patients should not remain on chemotherapy until radiological progression; instead, resectability should be periodically reassessed, and surgical resection or local ablative therapy should be considered once complete tumor clearance becomes technically achievable. Prolonged preoperative chemotherapy may increase the risk of chemotherapy‐associated liver injury and compromise surgical outcomes, while potentially reducing the opportunity for curative‐intent local treatment [245].

### 4.3 Ablation and SBRT within Multimodal Treatment

Radiofrequency ablation (RFA) is most appropriate for selected small lesions, particularly when surgical resection would require disproportionate loss of normal liver tissue or when comorbidities preclude major surgery. A needle with one or more electrodes is inserted into the tumor under ultrasound guidance. The electrodes generate high temperatures that induce cell death in the metastatic tissue [246]. In addition to its direct cytotoxic effects, RFA also exerts significant immune‐modulating effects that can enhance the body's antitumor response. One of the primary immunomodulatory effects of RFA is the activation of DCs, which is mediated through the upregulation of the expression of proinflammatory cytokines such as TNF‐α and IL‐1β. This activation promotes the priming and proliferation of CD4^+^T cells, thereby enhancing antitumor immune response [247]. In addition, RFA facilitates the release of tumor‐associated antigens and induces the expression of heat shock proteins, both of which are critical for antigen presentation and the recruitment of cytotoxic CD8^+^ T cells into the TME. RFA also increases NK cell activity by upregulating the expression of the activating receptor NKG2D, thereby potentiating innate immune‐mediated tumor clearance [248]. However, these immunological effects should not be equated with consistent systemic antitumor immunity in patients. Clinically meaningful abscopal responses remain uncommon and the principal determinant of local efficacy is complete tumor destruction with an adequate ablative margin [249].

Incomplete ablation (iRFA) represents a biologically distinct form of treatment failure. The hypoxic and inflammatory microenvironment created by iRFA promotes rapid tumor progression [250, 251]. Residual tumor burden following treatment impedes DC maturation, promotes the accumulation of immunosuppressive macrophages, and contributes to T‐cell depletion. These alterations collectively reshape the TME toward an immunosuppressive state, which reduces antitumor immunity and promotes resistance to ICI therapy, including PD‐1 blockade [252, 253]. To overcome these challenges, combination therapies are being explored. For example, in hepatocellular carcinoma, combining RFA with oncolytic peptide LTX‐315 and anti‐CTLA‐4 antibody [254], encapsulating nanoassemblies of anti‐PD‐L1 and spermidine‐grafted oxidized dextran [255], injectable polyoxometalate‐loaded coacervate delivery systems have been developed and shown promising results. In CRLM, similar multimodal approaches are being tested. Compared with RFA alone, combining RFA with neoadjuvant chemotherapy has yielded superior outcomes, including improved 3‐year local tumor progression‐free survival (PFS) and OS [256].

SBRT provides a noninvasive local option for patients who are unsuitable for surgery or RFA [257[]. ASCO guidance indicates that SBRT may be considered after systemic therapy in patients with liver oligometastases who are not candidates for resection [244] This sequence reflects the fact that systemic therapy addresses occult micrometastatic disease and reveals tumor biology, whereas SBRT consolidates control of persistent macroscopic lesions.

Local treatment may also be considered for patients with oligoprogressive disease, in which one or a small number of metastatic lesions progress while the remaining disease remains controlled by systemic therapy. In this context, ablation or SBRT may eradicate resistant subclones and permit continuation of otherwise effective systemic regimens. Nevertheless, this approach is largely based on selected patient data, and the optimal sequence of local treatment and subsequent systemic therapy remains under investigation. While SBRT provides effective local tumor control, disease progression outside the irradiated field—particularly the emergence of new liver metastases—remains the predominant mode of treatment failure [258, 259]. This underscores the need for integration with systemic therapies. Notably, patients who received post‐SBRT chemotherapy had significantly longer OS, supporting the rationale for combination approaches [260].

### 4.4 Immunotherapy in CRLM

Although standard treatment strategies for CRLM have notably improved patient survival, their efficacy remains limited in patients with unresectable disease and patients resistant to conventional therapies. These challenges underscore the urgent need for novel therapeutic strategies. In this context, advances in tumor immunology have paved the way for immunotherapy, which represents a promising approach that may overcome treatment resistance and offer new hope for difficult‐to‐treat patients. MSI/MMR status is the most important determinant of sensitivity to immune checkpoint blockade in patients with metastatic CRC.

#### 4.4.1 MSI‐H/dMMR CRLM

Compared with chemotherapy, first‐line treatment with pembrolizumab is advantageous for durable PFS in patients with MSI‐H/dMMR metastatic CRC (mCRC)[261], but a substantial fraction of patients with MSI‐H/dMMR CRC fail to respond to PD‐1/PD‐L1 blockade [243]. In contrast, in patients with MSS/pMMR mCRC, the objective response rates to single‐agent PD‐(L)1 blockade remain negligible, and emerging clinical evidence suggests that liver involvement itself can further reduce responsiveness to immunotherapy, with responses in some combination studies occurring predominantly in patients without liver metastases [262, 263]. Primary resistance in mCRC is driven by multiple interconnected mechanisms, including low tumor immunogenicity, impaired antigen presentation, limited T cell infiltration, suppressive myeloid populations, Tregs, abnormal vasculature, and fibroblast‐mediated stromal exclusion. The liver metastatic niche further reinforces these resistance programs through tolerogenic antigen presentation, immunoregulatory myeloid networks, and stromal signaling, which promote T‐cell dysfunction and exhaustion [264].

In addition to PD‐1/CTLA‐4 pathways, alternative inhibitory axes such as LAG‐3/FGL1 have attracted increasing attention [265]. Tumor‐infiltrating CD8^+^ T cells in patients with CRLM may express elevated levels of LAG‐3, while hepatocyte‐derived FGL1 can suppress CD8^+^ T‐cell and NK cell function, suggesting a potential mechanism of immune escape within the hepatic metastatic niche [68]. The LAG3‐targeting monoclonal antibody, relatlimab, combined with nivolumab is being investigated for the treatment of metastatic CRC (NCT05248048).

#### 4.4.2 MSS/pMMR CRLM

The situation is fundamentally different in patients with MSS/pMMR disease. Single‐agent PD‐1 or PD‐L1 blockade has minimal activity in MSS/pMMR mCRC, and liver involvement may further reduce therapeutic responsiveness. These constraints provide a rationale for intensification and rational treatment combinations. In preclinical MSS CRC models, dual blockade of CTLA‐4 and PD‐L1 produced stronger antitumor effects than either monotherapy did and markedly reduced liver metastatic burden, supporting the potential value of multicheckpoint inhibition therapy [266]. Next‐generation strategies that remodel “cold” MSS tumors, such as Fc‐enhanced CTLA‐4 blockade combined with PD‐1 inhibition, have shown encouraging activity in patients with relapsed MSS mCRC in early‐phase clinical studies, suggesting that qualitative reprogramming of immunity may be required in CRLM [267]. In addition, strategies integrating immunotherapy with antiangiogenic therapy have attracted interest because VEGF inhibition may normalize tumor vasculature, reduce myeloid suppression, and improve immune cell trafficking; consistently, adding atezolizumab to FOLFOXIRI plus bevacizumab improved outcomes in patients with first‐line mCRC, and research on biomarkers aims to clarify how liver metastases are beneficial in patients with pMMR disease [268, 269].

#### 4.4.3 Immunotherapy with Emerging Therapeutic Approaches

TTo improve response rates, researchers are exploring combination strategies with other therapies. The combination of ICIs with chemotherapy remains a cornerstone of treatment. Cytotoxic therapy is among the most established strategies combined with immunotherapy, although mechanistic examples should ideally be drawn from CRC‐ or CRLM‐relevant systems. In preclinical CRC settings, radioimmunotherapy combinations can increase intratumoral CD8^+^ T‐cell infiltration while reducing suppressive lymphocyte populations [270]. Radiotherapy also shows promise when combined with ICIs, increasing the efficacy of ICIs and generating abscopal effects, which suppress distant, unirradiated metastases. In CRC and CRLM models, combining radiotherapy with anti‐CD25/CTLA‐4 monoclonal antibodies increases CD8^+^ T cell infiltration and reduces the number of Tregs and exhausted T cells [271]. Notably, radiotherapy‐induced PAI‐1 promotes the transformation of pericytes into SFRP2‐high CAFs, which impair CD8^+^ T cell recruitment and suppress the abscopal effect. Targeting SFRP2 or PAI‐1 during radiation could improve outcomes when combined with ICI therapy [272].

Additional strategies under investigation include combining ICIs with agents not traditionally classified as frontline immunotherapies, such as low molecular weight heparin [273] and IL‐10 receptor alpha antibodies [274], which may improve prognoses for CRLM patients. The use of nanocarriers is another promising approach to improve drug delivery and the TME's response to ICIs. For example, a nanomedicine combining KIRA6, an α‐tocopherol nanoemulsion, and an anti‐PD‐1 antibody has been reported to restore antitumor immune responses in murine models, and an anti‐PD‐1 antibody can be used to rejuvenate CD8^+^ T cells and DCs, significantly increasing the efficacy of ICI therapy in patients with CRLM [275]. Similarly, Ding et al. developed a biomimetic nanodrug containing a cyclin‐dependent kinase inhibitor and PD‐L1 antibody, which increased responses to ICI therapy through similar mechanisms in CRLM patients [276].

Finally, as ICIs are intensified and combined, immune‐related adverse events (irAEs) become key feasibility constraints, with gastrointestinal and hepatic toxicities among the most consequential. Immune‐mediated gastrointestinal toxicity is among the most clinically consequential event, with higher rates generally observed with combination regimens than with PD‐1 or CTLA‐4 monotherapy [277]. Mechanistically, ICI‐induced colitis reflects a convergence of epithelial barrier disruption, myeloid infiltration and dysregulated tissue‐resident and circulating T‐cell activation, with the gut microbiome increasingly being implicated as a modulator of both susceptibility and recovery; this had motivated microbiome‐informed risk stratification aimed at preserving antitumor immunity [278]. Immune‐mediated hepatitis also presents unique challenges, as its incidence varies across studies [279]. In total neoadjuvant therapy studies incorporating sintilimab for rectal cancer, increased hepatotoxicity has been reported for some short‐course radiotherapy‐based regimens; however, whether this reflects the PD‐1 inhibitor, radiotherapy schedule, or intensified multimodal immune activation remains unresolved [280]. Compared with LCRT regimens, SCRT regimens may increase the risk of hepatotoxicity, possibly through enhanced antitumor immune activation. Future work should therefore focus on mechanistic investigations of irAEs, predictive biomarker development, and prospective multicenter evaluation across broader patient populations.

#### 4.4.4 CAR‐T

CAR‐T cell therapies have shown great promise in treating hematological malignancies, and recent efforts have aimed to extend these therapies to solid tumors, including CRC, particularly CRLM. Multiple clinical trials are investigating CAR‐T cells targeting specific tumor‐associated antigens such as NKG2D [281, 282], carcinoembryonic antigen (CEA) [283], LGR5 [284], and GUCY2C [285] in metastatic CRC.

Despite its success in hematologic malignancies, the efficacy of CAR‐T cell therapy in CRC—particularly in CRLM—is significantly hampered by the immunosuppressive TME. The liver's inherently tolerogenic nature is further compounded in metastatic settings by the accumulation of immunosuppressive cells and cytokines that impair CAR‐T cell function. For instance, CD11b^+^F4/80^+^ macrophages in liver metastases have been shown to induce apoptosis of CD8^+^ T cells through the Fas–FasL signaling axis [286], thereby reducing CAR‐T‐cell persistence and antitumor activity. To overcome these immunological barriers, several strategies are being explored. One approach involves the coadministration of proinflammatory cytokines such as IL‐15. Additionally, CAR‐T‐cell therapy can be combined with ICIs to reverse T‐cell exhaustion and enhance local immune activation. Encouragingly, early‐phase clinical trials utilizing IL‐15‐armored CAR‐T cells have demonstrated a disease control rate of 66% and an objective response rate of 33%, highlighting the potential of these combination approaches to improve therapeutic outcomes in patients with solid tumors such as CRLM [287].

Another challenge lies in the tumor heterogeneity between primary CRC and CRLM. Varied antigen expression can limit the ability of single‐antigen CAR‐T cells to effectively target all lesions [288]. To address this issue, dual‐targeting strategies, including tandem and bispecific CAR constructs, have been developed to recognize multiple tumor antigens simultaneously, increasing specificity and reducing the risk of antigen escape [289].

Furthermore, physical barriers within the CRLM, such as abnormal vasculature and dense stromal components, impair the trafficking and infiltration of CAR‐T cells to tumor sites. This problem is particularly pronounced in consensus molecular subtype (CMS) 4 CRC tumors, which are characterized by EMT, abnormal angiogenesis, and low immune cell infiltration, making them less likely to respond to CAR‐T therapy. Therefore, precise identification of subtypes of CRC or targeting these physical and molecular barriers, such as through molecular subtyping or the use of nanomedicine for improved CAR‐T delivery, may improve outcomes in these patients.

Finally, off‐target toxicity remains a significant concern. CAR‐T cells that target antigens that are also present on normal tissues can inadvertently cause severe unintended tissue damage. Therefore, increasing the specificity of CAR‐T‐cell constructs and incorporating safety switches are critical to reduce adverse effects [290].

#### 4.4.5 CAR‐NK

In contrast to CAR‐T‐cell therapy, CAR‐NK‐cell therapy has several advantages, including enhanced antitumor activity through multiple cytotoxic mechanisms and a reduced risk of irAEs [291, 292]. A small clinical trial of NKG2D CAR‐NK cells in patients with metastatic CRC showed promising results, with two of three patients demonstrating reduced tumor cell populations in ascitic fluid, while the third experienced rapid regression of liver metastases [293]. On the basis of these promising results, several clinical trials are currently investigating CAR‐NK therapies targeting specific colorectal tumor antigens, including EpCAM (NCT03013712), NKG2D (NCT05213195), and MUC‐1 (NCT02839954).

Despite these promising developments, CAR‐NK cells face challenges similar to those of CAR‐T cells, particularly limited infiltration into primary tumors and CRLM lesions, compounded by the immunosuppressive TME. Emerging targets, such as CD70, which is expressed in CRC, have attracted attention for CAR‐NK targeting. IL‐15 can increase CAR expression and promote the secretion of proinflammatory cytokines in CD70‐targeted CAR‐NK cells, increasing antitumor cytotoxicity [305]. Additionally, novel strategies, such as the use of adhesive hemostatic hydrogels loaded with STM2457, promote chemokine secretion, increasing CAR‐NK recruitment to the tumor site and reducing CRC recurrence [294].

#### 4.4.6 TIL

TIL therapy, a highly personalized form of ACT, involves isolating lymphocytes from the patient's tumor, expanding them ex vivo, and reinfusing them to induce an antitumor immune response. While this approach has shown potential, CRC is typically characterized by low TIL abundance, which poses challenges for both the collection and expansion of these lymphocytes [295]. To overcome this limitation, patient stratification is crucial to identify those with sufficient TILs or the potential for successful expansion. The Klintrup–Mäkinen score, a histopathological metric, is significantly correlated with increases in TIL levels derived from CRLM lesions [296].

Notably, tumor‐draining lymph nodes, which are rich in immune cells, are promising sources of lymphocytes for adoptive transfer. Tumor‐draining lymph nodes have TCR clonotypes similar to those of TILs from primary CRC tumors [297]. Moreover, lymphocytes derived from sentinel lymph nodes are effective in metastatic CRC patients even in smaller numbers when expanded under optimized conditions [298], suggesting that they could be a viable alternative source for TIL therapy. Innovations in culture systems have also improved TIL expansion. For example, Gretschel et al. used a perfusion bioreactor system supplemented with IL‐2 and IL‐12 to culture TILs from CRC tumor tissue, yielding a predominantly CD3^+^/CD8^+^ effector memory T‐cell population with strong cytolytic activity [299].

However, this approach was less effective in patients with advanced or metastatic CRC. To overcome this limitation, gene‐editing technologies—such as CRISPR–Cas9‐mediated PD‐1 knockout—have been employed to enhance the function of TILs. These strategies have demonstrated promising results, including a median 87.5% reduction in PD‐1 expression following TIL expansion, thereby enhancing TIL cytotoxicity and persistence [300]. These developments suggest that gene‐editing approaches could significantly improve the therapeutic potential of TIL‐based therapies in CRLM. Meanwhile, ongoing clinical trials continue to investigate immunotherapy in CRLM (Table 4).

**TABLE 4:** Exploring immunotherapy in colorectal cancer liver metastasis: a review of ongoing clinical trials.

Intervention	Target	Combination	Phase	References
Sintilimab	PD‐1/PD‐L1	PULSAR + standard systemic therapy	II	NCT06841159
Sintilimab	PD‐1/PD‐L1	SBRT + chemotherapy	III	NCT06794086
Sintilimab	PD‐1/PD‐L1	Cetuximab + chemotherapy	I/II	NCT06776757
Sintilimab	PD‐1/PD‐L1	Fruquintinib + chidamide	II	NCT06685276
Sintilimab	PD‐1/PD‐L1	MA + chemotherapy + bevacizumab or cetuximab	II	NCT06590259
Pembrolizumab	PD‐1/PD‐L1	Isunakinra	II	NCT06634875
Toripalimab	PD‐1/PD‐L1	Cetuximab + irinotecan	II	NCT06547203
Dostarlimab	PD‐1/PD‐L1	Chemotherapy	II	NCT06333314
Serplulimab	PD‐1/PD‐L1	Chemotherapy + bevacizumab	II/III	NCT06280495
Tislelizumab	PD‐1/PD‐L1	HAIC + targeted therapy	/	NCT06199232
JK08	CTLA‐4	/	I/II	NCT05620134
TSR‐022	TIM‐3	Nivolumab or TSR‐042 or docetaxel or pemetrexed + cisplatin/carboplatin	I	NCT02817633
CAR‐T	CDH17	/	I	NCT06937567
CAR‐T	CEA	/	I	NCT06821048
CAR‐T(C‐13‐60)	CEA	/		NCT06043466
CAR‐T((REVO‐UWD‐01))	/	/		NCT06653010
CAR‐T	LGR5	/	I/II	NCT05759728
CAR‐NK	NKG2D	/	I	NCT05213195
CAR‐macrophages	HER2	/	I	NCT04660929
TIL	/	Cyclophosphamide + fludarabine + IL‐2	II	NCT01174121
Dual Blockade of PD1/PDL1 and CTLA4	PD1/PDL1 + CTLA‐4	Antiangiogenic therapy	/	NCT06549907
Balstilimab + botensilimab	PD1/PDL1 + CTLA‐4	KRAS vaccine with poly‐ICLC adjuvant	I	NCT06411691
Balstilimab + botensilimab	PD1/PDL1 + CTLA‐4	Chemotherapy + bevacizumab	I/II	NCT05627635
Balstilimab + botensilimab	PD1/PDL1 + CTLA‐4	Dietary intervention + vitamin C	I	NCT06336902
Balstilimab + botensilimab	PD1/PDL1 + CTLA‐4	Regorafenib	I/II	NCT05672316
Botensilimab ± balstilimab	PD1/PDL1 + CTLA‐4	/	II	NCT05608044
Cadonilimab	PD1/PDL1 + CTLA‐4	Chemotherapy + bevacizumab	II	NCT05839470
SI‐B003	PD1/PDL1 + CTLA‐4	/	I	NCT04606472
Nivolumab + relatlimab	PD1/PDL1 + LAG‐3	/	III	NCT05328908
TIL + pembrolizumab	PD1/PDL1	Cyclophosphamide + fludarabine + IL‐2	I/II	NCT06530303
TIL + CRISPR	CISH	Cyclophosphamide + fludarabine + IL‐2	I/II	NCT04426669

Abbreviations: PULSAR: personalized ultrafractionated stereotactic adaptive radiotherapy; SBRT: stereotactic body radiation therapy; HAIC: hepatic arterial infusion chemotherapy.

Data sources: https://clinicaltrials.gov/.

## 5 Biomarkers for Risk Stratification, Prognosis, and Treatment Monitoring in CRLM

In CRLM, biomarkers are clinically meaningful when they provide biological information that cannot be fully captured by conventional radiological staging or clinicopathological parameters. Although advances in surgical techniques, systemic therapies, and imaging modalities have substantially improved outcomes for patients with CRLM, considerable heterogeneity remains among individuals with similar disease burdens and treatment strategies. Some patients achieve long‐term disease control after liver‐directed therapy, whereas others experience rapid disease recurrence despite apparently complete resection. This discrepancy reflects differences in tumor intrinsic biology, metastatic competence, the immune microenvironment, and residual disease burden, highlighting the need for biomarkers capable of improving patient stratification and guiding individualized treatment decisions.

### 5.1 Biomarkers for Predicting the Risk of Liver Metastasis

The development of liver metastasis is a complex biological process. Therefore, biomarkers predicting CRLM risk should ideally reflect not only tumor proliferation capacity but also metastatic fitness and the ability of tumor cells to adapt to the liver microenvironment.

Genomic alterations associated with aggressive tumor behavior represent important determinants of hepatic metastatic potential. Among genomic alterations, mutations affecting the RAS–RAF–MAPK pathway are among the most extensively investigated molecular determinants of metastatic behavior. KRAS mutations occur in approximately 40–50% of patients with CRC and contribute to constitutive activation of downstream signaling pathways involved in cell proliferation, invasion, angiogenesis, and EMT [301]. BRAF mutations, particularly the V600E variant, occur in several malignancies and are present in approximately 5% of patients with CRLM [302]. Compared with the KRAS mutation, the BRAF V600E mutation is associated with a worse prognosis and an increased risk of recurrence, and it appears to be a stronger adverse prognostic factor [303]. These molecular characteristics suggest that genomic profiling at the primary tumor stage may provide valuable information for identifying patients at increased risk of liver metastasis.

In addition to individual driver mutations, transcriptomic classifications have elucidated metastatic competence. The CMSs of CRC capture distinct biological programs associated with immune activation, stromal interaction, and epithelial plasticity. For most common distant metastases, liver metastases were predominantly CMS2 [304]. These characteristics suggest that transcriptomic biomarkers may complement genomic alterations in identifying patients at high risk for hepatic dissemination.

In addition, EV‐derived molecules are promising biomarkers that reflect the metastatic phenotype of CRC. Exosomes contain tumor‐derived nucleic acids, proteins, and regulatory RNAs that participate in intercellular communication and metastatic niche formation. Exosomal RNA signatures can distinguish CRC patients with liver metastatic progression. Lee et al. reported specific exosomal RNA patterns associated with CRLM progression, suggesting that exosomal profiling may provide a noninvasive approach for the early detection of metastatic potential [305]. Furthermore, cancer‐derived exosomal proteins actively promote liver metastasis by remodeling the hepatic microenvironment. For example, exosomal HSPC111 enhances CRLM through metabolic reprogramming of CAFs, indicating that exosomal molecules may function as both biomarkers and mechanistic indicators of metastatic evolution [48].

Similarly, exosomal circular RNAs represent potential biomarkers because of their stability and regulatory activity. Exosomal circPABPC1 promotes CRLM by regulating HMGA2 nuclear signaling and BMP4/ADAM19‐associated pathways, suggesting its potential value as a marker of metastatic competence [306].

### 5.2 Prognostic Biomarkers

After the development of CRLM, biomarkers are primarily used to estimate recurrence risk, survival probability, and the need for treatment intensification. The level of CEA remains a key biomarker in the management of CRLM, with well‐established prognostic significance. High pre‐LR and post‐LR serum CEA levels were significantly correlated with a poor prognosis in CRLM patients [307, 308]. In this context, high postoperative CEA can serve as a surrogate marker of minimal residual disease (MRD) missed on imaging, and postoperative CEA assessment may help refine the intensity and duration of adjuvant chemotherapy and surveillance. In addition, patients whose CEA levels fail to normalize during adjuvant therapy have significantly higher recurrence rates. These findings suggest that conventional clinicopathologic classifications may be inadequate for certain CRLM subgroups and that postoperative CEA levels may help identify patients who require closer follow‐up or intensified therapy [309]. The prognostic value of CEA levels can be further enhanced by considering the levels of cancer antigen 125, cancer antigen 19‐9, and other circulating biomarkers, enabling more dynamic assessments of tumor behavior [310, 311, 312].

Genomic biomarkers also provide important prognostic information after CRLM diagnosis. In the context of CRLM, mutant KRAS is associated with worse OS, and this adverse effect is more pronounced among patients who experience recurrence after CRLM resection [313]. Moreover, patients with KRAS‐mutated tumors may may benefit more from chemotherapy combined with targeted therapy than from chemotherapy alone [314]. These findings highlight that molecular classification should complement traditional clinicopathological parameters to improve prognostic assessment.

Nevertheless, the central challenge in curative‐intent CRLM management is MRD after apparently curative LR, which has accelerated the adoption of liquid biopsy approaches. Circulating tumor DNA (ctDNA) is increasingly considered the most informative blood‐based biomarker for patients with CRLM because ctDNA levels reflect tumor burden at the molecular level and can reveal molecular residual disease when conventional imaging is negative. Accordingly, multiple studies have shown that ctDNA levels can identify MRD in patients deemed disease‐free by imaging, enabling earlier and more biologically grounded risk stratification. Consistent with this concept, postoperative ctDNA positivity identifies patients with markedly increased recurrence risk and inferior survival, whereas ctDNA negativity after surgery, with or without perioperative systemic therapy, delineates patients with substantially more favorable long‐term outcomes, supporting ctDNA as an MRD surrogate and an early marker of treatment efficacy [315]. In parallel, data from resected CRLM cohorts show that postoperative ctDNA positivity aligns with poor pathological response and shorter recurrence‐free survival, underscoring that ctDNA can integrate residual microscopic viability across the hepatic metastatic compartment even when margins and imaging appear reassuring [316]. Serial postoperative ctDNA analysis has strong prognostic value and appears more sensitive for recurrence detection than standard‐of‐care CRLM surveillance tools, with reported lead times of up to 10.6 months [317].

### 5.3 Biomarkers of Treatment Response

Biomarkers for predicting treatment response are particularly important in patients with CRLM because therapeutic efficacy varies substantially among molecular and immune subgroups.

In terms of immunotherapy selection, MSI is among the most clinically mature immune biomarkers in patients with CRLM. Conceptually, MSI‐H tumors generate abundant neoantigens and an inflamed microenvironment, providing a strong rationale for checkpoint blockade; clinically, the durable activity of PD‐1‐based therapy in patients with MSI‐H mCRC is supported by long‐term follow‐up from first‐line pembrolizumab studies [318]. However, CRLM‐specific biology remains important: the liver is a tolerogenic immune organ, and multiple datasets across tumor types suggest that hepatic metastases can systemically and locally attenuate antitumor immunity, making it unclear whether MSI‐H status alone is sufficient to predict benefit when the dominant disease burden resides in the liver [319].

Tumor mutational burden (TMB) is mechanistically linked to MSI but may enable quantitative predictions when MSI alone fails to explain heterogeneous outcomes. In MSI‐H mCRC patients treated with checkpoint inhibitors, a higher TMB is associated with a response rather than progressive disease according to real‐world analyses. More broadly, TMB has been explored in dMMR/MSI‐H metastatic CRC cohorts to refine prognosis beyond MSI as a categorical variable [320, 321]. Importantly, for patients with CRLM, in whom most tumors are MSS, TMB also identifies a non‐MSI hypermutated subset, including tumors with POLE or POLD1 proofreading deficiency, that can be highly responsive to checkpoint blockade, thereby extending the biomarker concept from MSI‐defined immunogenicity to mutation‐driven immunogenicity, which may remain clinically actionable in the presence of liver metastases [322].

Liquid biopsy biomarkers are being increasingly investigated not only for prognosis but also for real‐time evaluation of treatment response. Dynamic changes in ctDNA levels during systemic therapy may reflect early molecular responses or the emergence of resistant clones before radiological progression. Therefore, integrating ctDNA monitoring with imaging and clinical parameters may enable the development of adaptive treatment strategies [323, 324].

In addition to ctDNA measurements, exosome‐based liquid biopsy represents an emerging strategy for monitoring the treatment of CRLM. Compared with ctDNA, exosomes have more stable structures and contain diverse molecular cargos, including microRNAs, circular RNAs, and proteins. These molecules may provide information regarding tumor evolution, immune regulation, and therapeutic resistance. Exosomal RNA signatures are associated with CRC progression and liver metastatic behavior, supporting their potential application as complementary biomarkers for treatment monitoring [325]. However, compared with ctDNA‐based methods, exosome‐based approaches must be clinically validated and assessed through standardized analytical workflows before they are routinely applied.

## 6 Conclusion

### 6.1 CRLM Represents a Dynamic Process Driven by Tumor–Liver Crosstalk

CRLM remains a major cause of treatment failure and mortality in CRC. Although portal venous drainage provides an anatomical explanation for the frequent involvement of the liver, metastatic colonization is determined by much more complex biological processes. Disseminated CRC cells must overcome multiple barriers, including detachment from the primary tumor, survival in circulation, vascular arrest, extravasation, and adaptation to the hepatic niche. Only a small proportion of tumor cells with appropriate genetic, epigenetic, and phenotypic characteristics can complete this process.

Current evidence indicates that CRLM is not driven solely by intrinsic alterations in tumor cells. Instead, metastatic progression depends on continuous interactions between CRC cells and the liver microenvironment. The physiological immune tolerance of the liver and the activity of HSCs, endothelial cells, and resident immune cells create conditions that facilitate CRC cells survival and expansion. Furthermore, primary CRC can actively prepare the liver before metastasis through EVs, soluble factors, inflammatory signals, and alterations in the gut–liver axis. These observations support the view that metastatic progression begins before liver lesions become clinically detectable. Defining these early tumor–liver interactions may therefore improve risk stratification and identify opportunities for earlier intervention.

### 6.2 Immune Suppression and Microenvironmental Remodeling Are the Key Barriers to Effective CRLM Treatment

Lymphocyte dysfunction in CRLM arises from multiple and context dependent mechanisms. Checkpoint signaling, impaired antigen presentation, and immunosuppressive cytokines act alongside metabolic and stromal constraints in the liver to restrict lymphocyte recruitment, activation, and cytotoxic function. Together, these changes impair CD8^+^ T‐cell and NK‐cell function, weaken antitumor immune surveillance, and favor metastatic progression

Immune suppression in CRLM cannot be attributed to a single cell population or signaling pathway. Rather, it results from interactions among Tregs, MDSCs, TAMs, KCs, HSCs, endothelial cells, and components of the ECM.

These mechanisms may help explain the immunologically “cold” phenotype of CRLM. Moreover, the density and functional state of lymphocytic infiltration, together with systemic inflammatory indices such as the neutrophil‐to‐lymphocyte ratio and lymphocyte‐to‐monocyte ratio, are increasingly recognized as prognostic and potentially predictive signals. Together, these observations suggest that immune profiling could complement conventional clinicopathological staging and improve risk stratification in CRLM.

### 6.3 Unresolved Questions in CRLM Biology and Clinical Translation

Despite substantial progress in understanding CRLM biology, several important questions remain remain unresolved. First, the mechanisms by which only specific CRC subclones acquire liver metastatic capacity remain incompletely understood. Although genomic alterations involving the KRAS, APC, TP53, WNT/β‐catenin, PI3K/AKT, and TGF‐β signaling pathways are associated with metastatic behavior, how genetic evolution interacts with epigenetic plasticity, immune selection, and liver‐specific adaptation remains unclear.

Second, the temporal and spatial dynamics of metastatic niche formation remain poorly characterized. The transition from PMN formation to early colonization remains difficult to capture. A better understanding of these early events may help in the identification of therapeutic windows for preventing metastasis.

Third, although numerous potential biomarkers and therapeutic targets have been identified, their clinical implementation remains limited. Their translation is complicated by marked variation among patients in tumor genetics, immune composition, metabolic state, and treatment response. Biomarkers that combine several of these features may therefore be more informative than single markers for patient stratification.

Progress will also depend on experimental models that more faithfully reproduce hepatic immunometabolism, vascular organization, and stromal remodeling. Single‐cell sequencing, spatial profiling, and integrated multiomics approaches should help resolve how CRLM evolves across time, space, and treatment and may identify alterations that can be tested as biomarkers or therapeutic targets.

### 6.4 Future Perspectives Toward Precision Management of CRLM

Treatment of CRLM has increasingly combined systemic chemotherapy, targeted agents, hepatic resection, local ablation, and radiotherapeutic approaches. Despite these advances, recurrence and treatment resistance remain common.

Further improvement will require better treatment sequencing and combination strategies informed by both tumor biology and the metastatic microenvironment. Major clinical goals include increasing the proportion of patients who can undergo potentially curative resection and reducing early recurrence after surgery through molecular and immune stratification.

The biology of lymphocyte dysfunction also suggests several therapeutic opportunities in CRLM. Approaches aimed at enhancing intratumoral immune recruitment, such as chemokine engineering, increasing tumor antigenicity through oncolytic platforms, restoring effector function through combinations of immune checkpoint blockade with agents targeting metabolic or other suppressive pathways. Similarly, adoptive cellular therapies, including CAR‐T‐based and TIL‐based approaches, may require modulation of hepatic immune tolerance to achieve durable activity in patients with CRLM.

Advanced imaging, ctDNA, and circulating immune signatures may help monitor treatment response, detect MRD, and refine perioperative risk assessment. Ultimately, biomarker‐guided and personalized strategies can be developed only through the integration of epidemiology, molecular pathogenesis, immune ecology, and clinical management to improve resectability, reduce recurrence, and extend survival for patients with CRLM.

## Author Contributions

LF, HZZ, and JLF contributed to conception of the study. JLF and HRZ drafted the manuscript. DYK, YS, QZ, RSX, SBL, WP, and WJY provided suggestions for revision. LF and HZZ revised the manuscript. All authors read and approved the final manuscript.

## Funding

This work was supported by the National Key Research and Development Program of China (2022YFF0710603) and the Chinese Academy of Medical Sciences Initiative for Innovative Medicine (2021‐I2M‐1‐018, 2023‐I2M‐2‐004).

## Ethics Statement

The authors have nothing to report.

## Conflicts of Interest

The authors declare no conflicts of interest.

## Data Availability

No datasets were generated or analyzed during the current study.
